# Free‐Standing Halogen‐Bonded Nanosheets Formed by Ultrasonic Liquid Exfoliation

**DOI:** 10.1002/advs.202507800

**Published:** 2025-10-29

**Authors:** Prioti Choudhury Purba, Elisa Marelli, Thomas M. Roseveare, Joshua Nicks, Lee Brammer, Nicholas G. White, Natalia Martsinovich, Giuseppe Resnati, Pierangelo Metrangolo, Jonathan A. Foster

**Affiliations:** ^1^ School of Mathematical and Physical Sciences University of Sheffield Sheffield S10 2TN UK; ^2^ Department of Chemistry, Materials, and Chemical Engineering “Giulio Natta” Politecnico di Milano Via L. Mancinelli 7 Milan 20131 Italy; ^3^ Research School of Chemistry The Australian National University Canberra ACT 2601 Australia

**Keywords:** 2D material, halogen bond, nanosheet, supramolecular, ultrasonic liquid exfoliation

## Abstract

Strong in‐layer interactions are typically considered a key requirement for exfoliating layered materials to form nanosheets. Here, the formation of free‐standing nanosheets held together by halogen bonding interactions is reported for the first time. Layered co‐crystals are synthesized using either iodo‐ or bromo‐functionalized halogen bond donors with pyridyl functionalized acceptors. Sonication resulted in dissolution in a range of polar organic solvents; however, stable suspensions showing Tyndall scattering are observed for the iodo‐systems after exfoliation in water. Atomic force microscopy confirms the formation of micron‐sized nanosheets approaching monolayer thickness. Powder X‐ray diffraction of the nanosheets shows preferred orientation along the (1 1 0) plane, which confirms that the nanosheets are composed of halogen‐bonded chains in one dimension, and *π*–*π* stacking interactions in the second. The iodo‐nanosheets are stable in acidic and alkaline conditions and when heated at 80 °C for 48 h. Comparisons between the iodo‐ and bromo‐ analogues confirm that stronger halogen bonding is likely responsible for the difference in stability. The remarkable stability shown by these nanosheets held together by only supramolecular interactions calls for re‐evaluation of the conventional understanding of the requirements for forming 2D materials and establishes halogen‐bonded nanosheets as an exciting new class of materials.

## Graphical abstract

Free‐standing nanosheets held together by halogen bonding interactions (XB), formed through ultrasonic liquid exfoliation of layered molecular crystals, are reported here for the first time. The work challenges the conventional requirement for strong in‐layer interactions for the formation of stable nanosheets through liquid exfoliation and establishes halogen‐bonded nanosheets (XONs) as an exciting new class of nanomaterials.

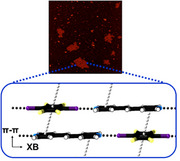

## 1 Introduction

2D materials have emerged as an important class of nanomaterials owing to their remarkable mechanical, electronic, optical, and magnetic properties.^[^ ^1^, ^2^ ^]^ Ultrasonic liquid‐phase exfoliation is a simple and scalable approach to converting a wide range of layered materials into 2D nanosheets.^[^ ^3^, ^4^, ^5^ ^]^ This process uses ultrasound energy to overcome weak inter‐layer interactions without disrupting stronger in‐layer interactions in order to produce free‐standing mono‐ or few‐layered nanosheets with high aspect ratios.^[^ ^6^, ^7^ ^]^ This approach has therefore typically been applied to inorganic materials that are held together through strong covalent bonds, such as in graphite,^[^ ^8^ ^]^ boron nitride,^[^ ^9^, ^10^ ^]^ and materials such as layered oxides,^[^ ^11^ ^]^ clays, and zeolites.^[^ ^12^ ^]^ More recently, layered molecular materials formed through dynamic covalent or coordination bonds have been used to produce covalent organic framework nanosheets (CONs)^[^ ^13^, ^14^, ^15^ ^]^ or metal‐organic framework nanosheets (MONs).^[^ ^7^, ^16^, ^17^, ^18^, ^19^ ^]^ The more dynamic bonding present in these materials allows them to be produced under milder conditions, and their molecular nature allows their structure and properties to be more easily modified than for inorganic nanosheets. This combination of properties makes them ideal for a wide range of sensing,^[^ ^20^, ^21^ ^]^ catalysis,^[^ ^22^, ^23^ ^]^ electronics,^[^ ^24^, ^25^ ^]^ and separation^[^ ^26^, ^27^ ^]^ applications.

We recently demonstrated for the first time how ultrasonic liquid exfoliation of small molecule organic compounds can be used to form hydrogen‐bonded organic nanosheets (HONs).^[^ ^28^ ^]^ The micron‐sized monolayered nanosheets are held together by strong charge‐assisted carboxylate‐amidinium hydrogen bonds and show remarkable stability even after heating for several days in water. Other examples of HONs held together by neutral hydrogen bonds have since been reported.^[^ ^29^, ^30^, ^31^ ^]^ For example, ultrasonic liquid exfoliation of hydrogen‐bonded frameworks (HOFs) based on guanine‐quadruplex moieties and duplex‐hydrogen bonds has produced ultrathin HONs held together by neutral hydrogen bonds. In another example, free‐standing monolayered nanosheets were developed by exfoliating crystals of a discrete supramolecular coordination complex where metallacycles were held together by multiple weak *π*–*π* stackings and CH···π interactions.^[^ ^32^ ^]^ These counterintuitive results go against the common perception that strong in‐layer interactions are needed to form stable nanosheets, and they led us to question whether liquid exfoliation could be used to form nanosheets held together by other supramolecular in‐layer interactions.

Halogen bonding has become a significant tool for crystal engineering, and the use of this intermolecular interaction has opened up new avenues for the design and synthesis of supramolecular materials.^[^ ^33^, ^34^, ^35^, ^36^, ^37^, ^38^ ^]^ Formed between a region of positive electrostatic potential on a halogen (X) and the lone pair of a Lewis base, halogen bonds display a distinct set of properties compared to other supramolecular interactions.^[^ ^39^ ^]^ Halogen bond angles are highly directional, with a linear connection made between donor and acceptor groups.^[^ ^40^ ^]^ The strength of halogen bonds can range between 10–200 kJ mol^−1[^ ^41^, ^42^ ^]^ and can be systematically tuned by varying the polarizability of the donor halogen (I > Br > Cl >> F) and strengthened by connecting to strongly electron‐withdrawing groups such as perfluorocarbons.^[^ ^43^ ^]^ The halogenated organic fragments interact only weakly with hydrogen bond donors, making them hydrophobic and often stable in water.^[^ ^44^ ^]^ Halogen bonds have been harnessed to form a diverse range of supramolecular structures ranging from cages and ion transporters^[^ ^45^, ^46^ ^]^ to porous frameworks,^[^ ^47^, ^48^ ^]^ photoresponsive materials,^[^ ^49^, ^50^ ^]^ films,^[^ ^51^, ^52^, ^53^ ^]^ liquid crystals,^[^ ^54^, ^55^ ^]^ and gels.^[^ ^56^ ^]^ These materials can be formed at low temperatures in high yields and have been employed in a wide range of sensing,^[^ ^57^, ^58^ ^]^ catalytic,^[^ ^59^, ^60^ ^]^ separation,^[^ ^61^ ^]^ and drug design^[^ ^62^, ^63^ ^]^ applications.

A variety of halogen bond‐induced 2D assemblies on surfaces have been reported,^[^ ^64^, ^65^ ^]^ however to our knowledge, none have then been removed from their supports to form free‐standing sheets. Self‐assembled membranes containing halogen bonds have also been reported, but assembly in these structures is typically driven by other interactions, such as the hydrophobic effect, with halogen bonds then shielded from direct interactions with solvent molecules.^[^ ^36^, ^66^, ^67^, ^68^ ^]^ A very large number of crystalline structures containing halogen bonds have been reported so far. As an example, the latest release of the Cambridge Structural Database (CSD 5.46) contains 1279 high‐quality crystal structures showing directional N···I─C interactions, and 681 showing N···Br─C ones, which are all relevant to the present study.^[^ ^69^ ^]^ Many of them form layered structures alongside other supramolecular interactions such as hydrogen bonds, *π*–*π* stacking, and van der Waals interactions. However, to the best of our knowledge, none have been exfoliated to form free‐standing halogen‐bonded nanosheets (XONs).

Herein, we report the first examples of ultrasound‐assisted liquid exfoliation of halogen‐bonded layered crystals to form free‐standing nanosheets. Bromo‐ and iodo‐analogues of a co‐crystal were synthesized and exfoliated in water. The nanoscopic dimensions, molecular structure, and stability of the resulting materials were compared, and differences in bonding were analyzed by DFT calculations. These results help redefine our understanding of the requirements of bonding within 2D materials and provide the first examples of an exciting new class of materials.

## 2 Results and Discussion

### 2.1 Synthesis of Layered Halogen‐Bonded Co‐Crystals

**Scheme 1:**
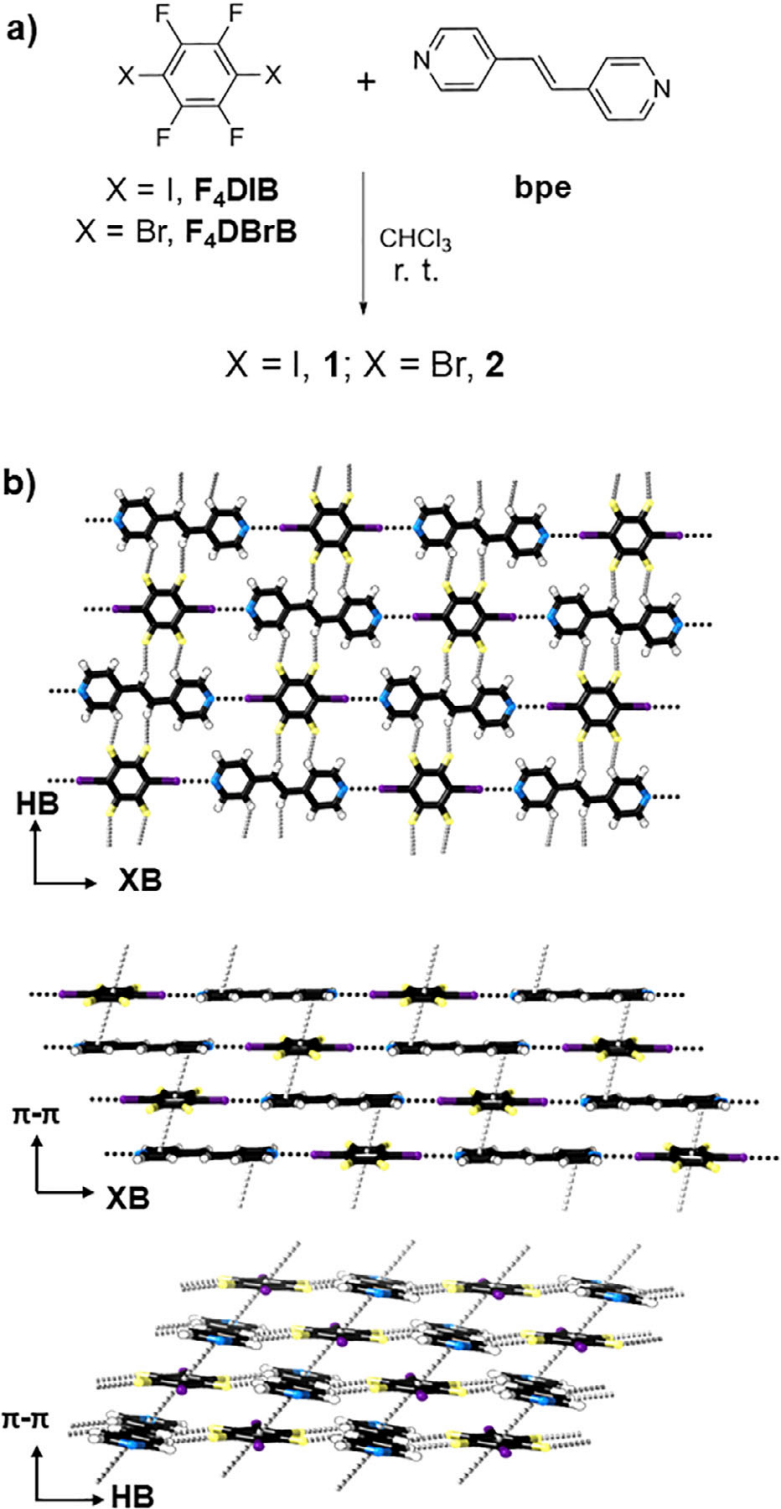
a) Reaction scheme for the synthesis of **1** and **2**. b) Crystal structures of **1** showing different combinations of halogen bonding (XB), hydrogen bonding (HB), and *π*–*π* stacking interactions. Halogen and hydrogen bonds shorter than the sum of the van der Waals radii and aromatic contacts with centroid∙∙∙centroid distances less than 4 Å are shown with dotted lines. Color codes: C (black), H (white), N (blue), F (yellow), and I (purple).

**Figure 1:**
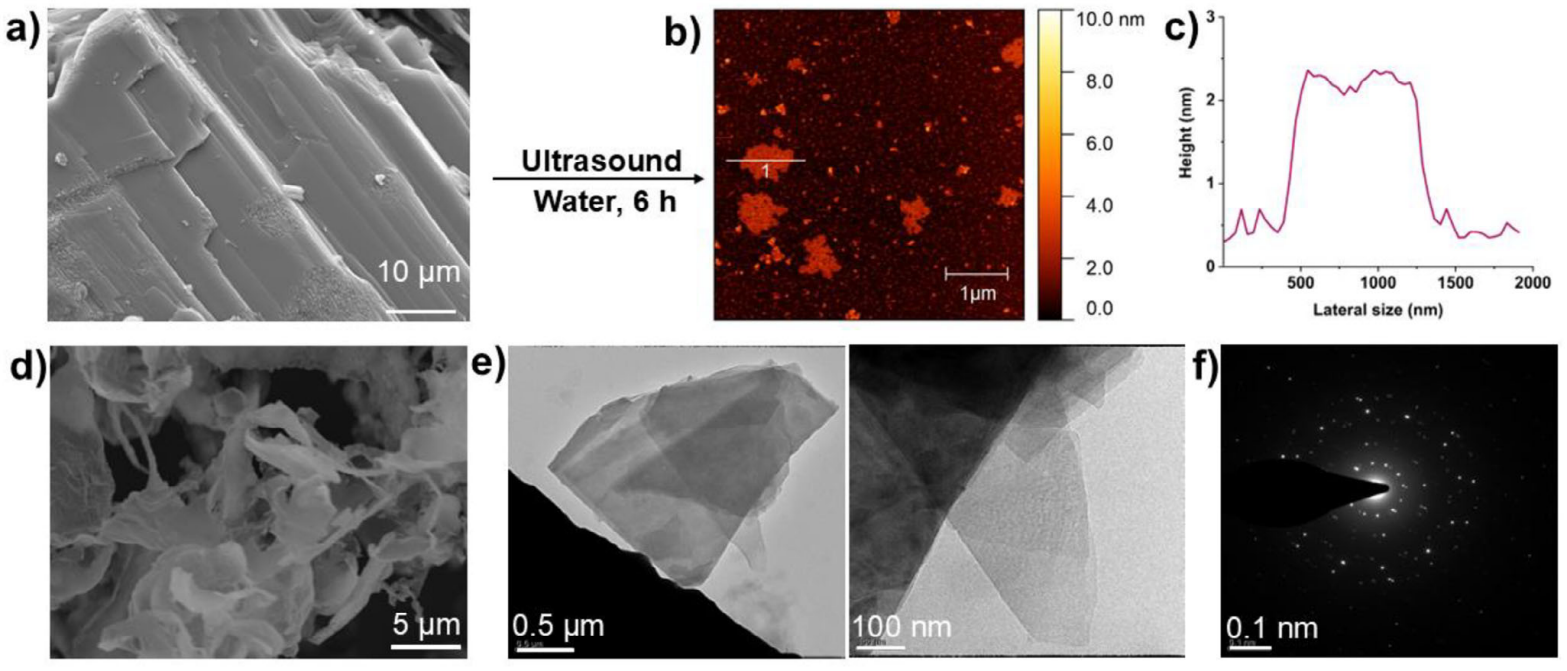
Ultrasonic liquid exfoliation of a) layered co‐crystal **1** in water medium to form b) nanosheet **XON1**. c) Height profile of the nanosheet. d) SEM image of the freeze‐dried **XON1**. e) TEM images and f) Electron diffraction of nanosheet **XON1**.

**Figure 2:**
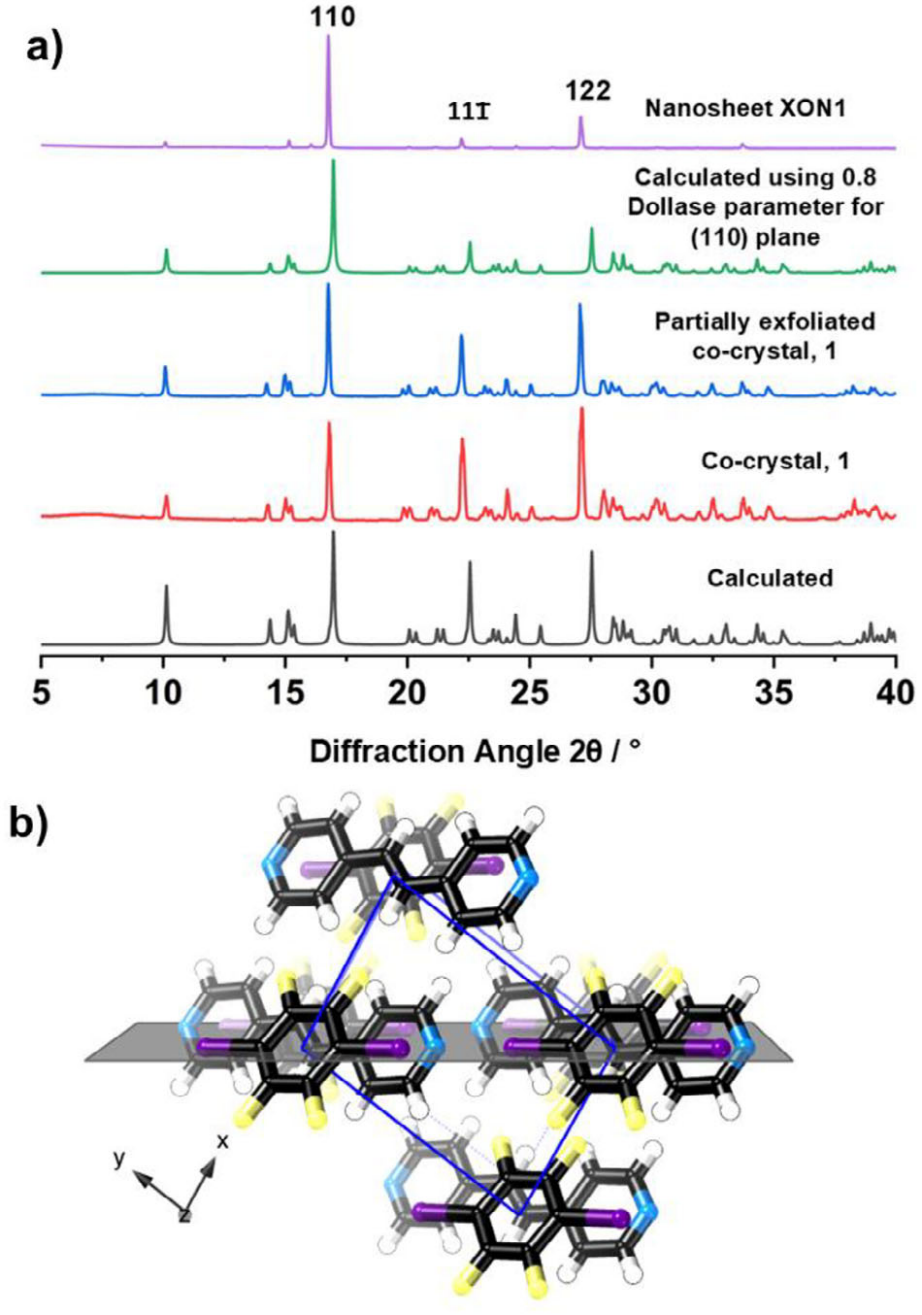
a) Powder X‐ray diffraction patterns for **1**. From bottom to top: the calculated pattern from CSD 245 125 (ref code: QIHCAL06), as‐synthesized co‐crystal, partially exfoliated co‐crystal recovered from water, calculated pattern with Dollase parameter of 0.8 applied along the (1 1 0) plane to imitate preferred orientation by the nanosheets when deposited onto the sample holder, and pattern for **XON1** nanosheets recovered from water. b) View of the (1 1 0) plane of the X‐ray crystal structure of **1** (grey), the unit cell is shown in blue. Color codes: C (black), H (white), N (blue), F (yellow), and I (purple).

Two different layered co‐crystals, **1** and **2** (**Scheme** **1**), were synthesized according to a previously reported procedure.^[^ ^70^, ^71^, ^72^ ^]^ A halogen bond donor, either 1,4‐diiodotetrafluorobenzene (**F_4_DIB**) or 1,4‐dibromotetrafluorobenzene (**F_4_DBrB**), as well as the halogen bond acceptor 1,2‐bis(4‐pyridyl)ethylene (**bpe**), were dissolved in CHCl_3_ in a 1:1 ratio, and the solvent slowly evaporated to yield colourless crystals. Analysis of the samples by powder X‐ray diffraction (PXRD) showed a good match between the calculated patterns from the reported crystal structures of iodo‐containing compound **1** and bromo‐containing compound **2** (Figures 2; Figures S3 and S5, Supporting Information). Fourier‐transform infrared (FTIR) spectroscopy and elemental analysis results were in agreement with previously reported data.^[^ ^70^, ^71^ ^]^ SEM imaging indicated the presence of layers within both the micro‐crystalline powders of **1** and larger crystals obtained for **2** (**Figure** **1**a; Figures S8 and S9, Supporting Information).

Analysis of previously obtained X‐ray crystal structures for **1** (Scheme 1) and **2** (Figure S7, Supporting Information) shows they consist of 1D halogen‐bonded chains formed by alternating donor and acceptor molecules, labeled as the XB direction in Scheme 1, with N∙∙∙X distances of 2.77 and 2.82 Å for N∙∙∙I and N∙∙∙Br, respectively. These chains are connected together in a second dimension (labeled as the HB direction) through H···F hydrogen bonds, with bond lengths ranging between 2.41 and 2.52 Å. The third orthogonal direction consists of alternating stacks of offset donor and acceptor molecules (labeled *π*–*π*). These are more offset in the case of **2,** such that distances between **F_4_DBrB** and **bpe** ring centroids are further in this co‐crystal (4.30 Å) than in **1** (**F_4_DIB**∙∙∙**bpe** centroid∙∙∙centroid distance = 3.89 Å), but **2** features relatively close contacts between H or F atoms and the ring centroid (3.33 and 3.27 Å, respectively).

The combination of a strong pyridyl halogen bond acceptor with iodine or bromine connected to perfluorocarbons as halogen bond donor results in strong halogen bonding interactions within this system. DFT calculations from a previous report show that the bond dissociation energy of iodine‐containing **1** is 6.02 kcal mol^−1^ and, for a similar system to **2** (co‐crystals of 1,2‐bis(4‐pyridyl) ethane with **F_4_DBrB**), the bond dissociation energy is lower at 3.65 kcal mol^−1^.^[^ ^72^ ^]^ Another study with related systems shows that C─H ···F interactions in such systems are quite weak (≈1 kcal mol^−1^),^[^ ^73^ ^]^ indicating that although three types of interactions are present in our layered systems, halogen bond formation is the primary driving force for the self‐assembly process.

### 2.2 Preparation of Halogen‐Bonded Nanosheets (XONs) by Liquid Exfoliation

Exfoliation was carried out using a previously reported sonicator setup (see Section S3, Supporting Information) in which samples were subjected to “soft” 80 kHz ultrasound in a temperature‐controlled ultrasonic bath and rotated continuously using an overhead stirrer to minimize hot‐spots and aid reproducibility.^[^ ^16^, ^74^, ^75^ ^]^ In contrast to nanosheets based on covalent bonds, which are insoluble in most liquids, a key requirement for forming nanosheets from molecular crystals is that the components remain insoluble in the exfoliation solvent. A range of solvents were therefore investigated to check the solubility of the crystals and identify suitable candidates for exfoliation. Compound **1** was found to dissolve immediately after the addition of low polarity solvents such as toluene, diethyl ether, dichloromethane, and dioxane. In polar solvents such as acetone, methanol, acetonitrile, and DMF, the sample partially dissolved on the addition of the solvent, with no material remaining after 1 h of sonication. Samples of **2** were dissolved immediately in all the aforementioned solvents. However, in water, both **1** and **2** showed strong Tyndall scattering when a laser was shone through the samples after 1 h of sonication. Halogen bonds have been successfully used to create a wide range of stable supramolecular assemblies in water and are often considered to be hydrophobic compared to hydrogen bond tectons because their building blocks are more lipophilic.^[^ ^33^ ^]^

The following optimized process was used for the exfoliation of all samples. Layered co‐crystals of **1** or **2** (5 mg) were suspended in 6 mL of water and sonicated in an ultrasound bath at 80 kHz for 6 h at 20 °C. The resulting suspensions were then centrifuged at 600 rpm for 10 mins, followed by another 10 mins of centrifugation at 800 rpm to remove unexfoliated materials. The resulting transparent suspensions showed Tyndall scattering, indicating the presence of nanoparticles (Figures S10 and S26, Supporting Information).

Particle size analysis was undertaken through AFM imaging of suspensions deposited onto mica using a hot‐drop method.^[^ ^28^ ^]^ Images of the iodo‐system, **1**, revealed the formation of high aspect‐ratio nanosheets, **XON1** (Figure 1b; Figures S11 and S12, Supporting Information). A statistical analysis (*n* = 54) of the nanosheet thickness was undertaken, with the majority (38) showing a uniform thickness of 1.8 nm, with the rest measured as up to 2.1 nm thick against the background (Figure S12, Supporting Information). The lateral dimensions of the nanosheets showed a range of 300–1500 nm, giving them an aspect ratio ≈500. This is consistent with dynamic light scattering measurements of the colloidal suspension of **XON1,** which show a mean particle size ≈830 nm (Figure S13, Supporting Information). Since the expected thickness of a single layer of nanosheets based on the crystal structure for **1** is 0.5 nm, the AFM measurements might be interpreted to indicate the formation of 3–4 layer thick nanosheets. Although few‐layer nanosheets are possible, we see no obvious reason why bi‐ tri‐ or tetra‐layer nanosheets would be formed so consistently, with no thinner sheets or step‐edges observed. AFM measurements often overestimate nanosheet thickness,^[^ ^76^ ^]^ and we suggest these data are consistent with exfoliation down to monolayer sheets.

A suspension of the nanosheets in water was freeze‐dried, and the resulting nanosheet powder was imaged by SEM. An ultrathin sheet‐like morphology (Figure 1d; Figure S14, Supporting Information) was obtained from SEM images, which is different from the layered morphology of the co‐crystal **1** (Figure 1a; Figure S8, Supporting Information). TEM images of suspensions of **XON1** drop‐casted onto 200‐mesh carbon film‐coated TEM grids also confirmed the presence of few‐layer nanosheets, which are ≈2 µm in size (Figure 1e). Selected area electron diffraction of the same sample clearly shows visible bright spots, indicating the nanosheets maintain their crystallinity even after exfoliation (Figure 1f).

Powder X‐ray diffraction patterns (PXRD) for the isolated nanosheets and bulk material following exfoliation were collected to check the phase of the resulting material. The PXRD pattern for the partially exfoliated bulk material matched well with the as‐synthesized co‐crystal and the calculated pattern obtained from the reported crystal structure, which indicates the extended crystalline structure of **1** was maintained after sonication (**Figure** **2**a; Figure S18, Supporting Information). Pawley fitting of patterns for isolated **XON1** nanosheets confirmed they also match the expected structure (Figure S17, Supporting Information), although significant changes are observed in the intensity of peaks relative to those of the as‐synthesized co‐crystal. This is attributed to preferred orientation, which is common for nanosheets (vide infra).

Liquid exfoliation typically relies on strong interactions within layers of a crystal to maintain the integrity of the nanosheets whilst separating apart the layers. However, the bulk co‐crystals of **1** contain relatively weak interactions in all three dimensions, so it is not necessarily obvious which dimension will be broken. The crystals could either be “sliced” through the *π*–*π* interactions to produce XB‐HB nanosheets, through the hydrogen‐bonds to produce XB‐π nanosheets, or through the halogen bonds to produce HB‐π nanosheets (as shown in Scheme 1).

PXRD patterns for **1** were therefore simulated with Dollase parameters,^[^ ^77^ ^]^ which define the extent of preferred orientation, applied along the [1 2 2], [1 1 0] and [0 1 0] vectors, corresponding to normal to the XB‐HB, XB‐π and HB‐π nanosheet planes, respectively (Figure S16, Supporting Information). The closest match was found when a Dollase parameter of 0.8 was applied along the [1 1 0] vector (Figure 2b; see Figure S15, Supporting Information for full analysis). The (110) plane is coincident with the XB‐π plane of the nanosheets (Figure 2b), indicating that, in water, the hydrogen bonds break, leaving nanosheets held together by the relatively hydrophobic halogen bonding and *π*–*π* interactions.

These results are supported by DFT calculations, which were used to evaluate the strength of interactions in different directions within the crystals. The PBE functional^[^ ^78^ ^]^ with the D3 dispersion correction^[^ ^79^ ^]^ was used to calculate relative binding energies for dimers (one halogen bond donor–acceptor pair relative to the separated molecules), 1D chains along the halogen bonding, hydrogen bonding, and *π*–*π* stacked directions, 2D nanosheets (XB‐HB, XB‐π and HB‐π) and 3D co‐crystals for both **1** and **2**, which are summarized in **Table** **1**. Binding in the XB and *π*–*π* directions for **1** was found to be of similar strength at −16.8 and −19.1 kcal mol^−1^, whilst HB is significantly weaker at −7.7 kcal mol^−1^. This then translates into XB‐π being the energetically preferred nanosheets at −47.0 kcal mol^−1^, compared to −32.2 and −30.3 kcal mol^−1^ for the putative XB‐HB and HB‐π nanosheets, respectively. These results are therefore consistent with what is observed from PXRD experiments, that the crystals are sliced through the HB direction on exfoliation to give the XB‐π nanosheets. The similarity in energy between the XB and *π*–*π* directions is also reflected in the relatively isotropic nanosheets observed, rather than more tape‐like structures, which would be expected if bonding were substantially stronger in one direction than the other. It is also worth noting that the binding energies within **XON1** are significantly weaker than those calculated for our previously reported HONs^[^ ^28^ ^]^ based on amidinium‐carboxylate moieties forming 2D hydrogen‐bonding networks, which were calculated to have in‐plane HB binding energies of −72.1 and −55.6 kcal mol^−1^.

**Table 1:** Calculated binding energies for different components based on experimental crystal structure.

	Relative binding energy [kcal mol^−1^]	
System	1	2
Halogen‐bonded dimer	−8.8	−5.3
Halogen‐bonded chains	−16.8	−10.6
Hydrogen‐bonded chains	−7.7	−6.8
*π*–*π* stacking chains	−19.1	−19.9
Halogen + hydrogen bonded sheet (XB‐HB)	−32.2	−22.6
Halogen + *π*–*π* stacking sheet (XB‐π)	−47.0	−31.2
Hydrogen + *π*–*π* stacking sheet (HB‐π)	−30.3	−32.3
3D co‐crystal	−69.9	−53.5

Synthetic yields of 7–12% were obtained for **XON1**, which are high compared to most other existing micron‐sized monolayered supramolecular nanosheets.^[^ ^80^, ^81^ ^]^ UV–vis spectroscopy of **XON** **1** shows a distinct absorption pattern compared to those of its constituent parts, **bpe** and **F_4_DIB** (Figure S20a, Supporting Information). When suspensions of **XON1** were centrifuged at 6000 rpm to remove all nanosheets, UV–vis measurements of the resulting solution showed trace amounts of dissolved **bpe** in solution (Figure S20b, Supporting Information). When suspensions of **XON1** were drop‐cast from solution, no evidence of the growth of needle‐like crystals of **bpe‐H_2_O** was observed (Figure S22, Supporting Information), providing further evidence for the low concentration of dissolved **bpe**.

**Figure 3:**
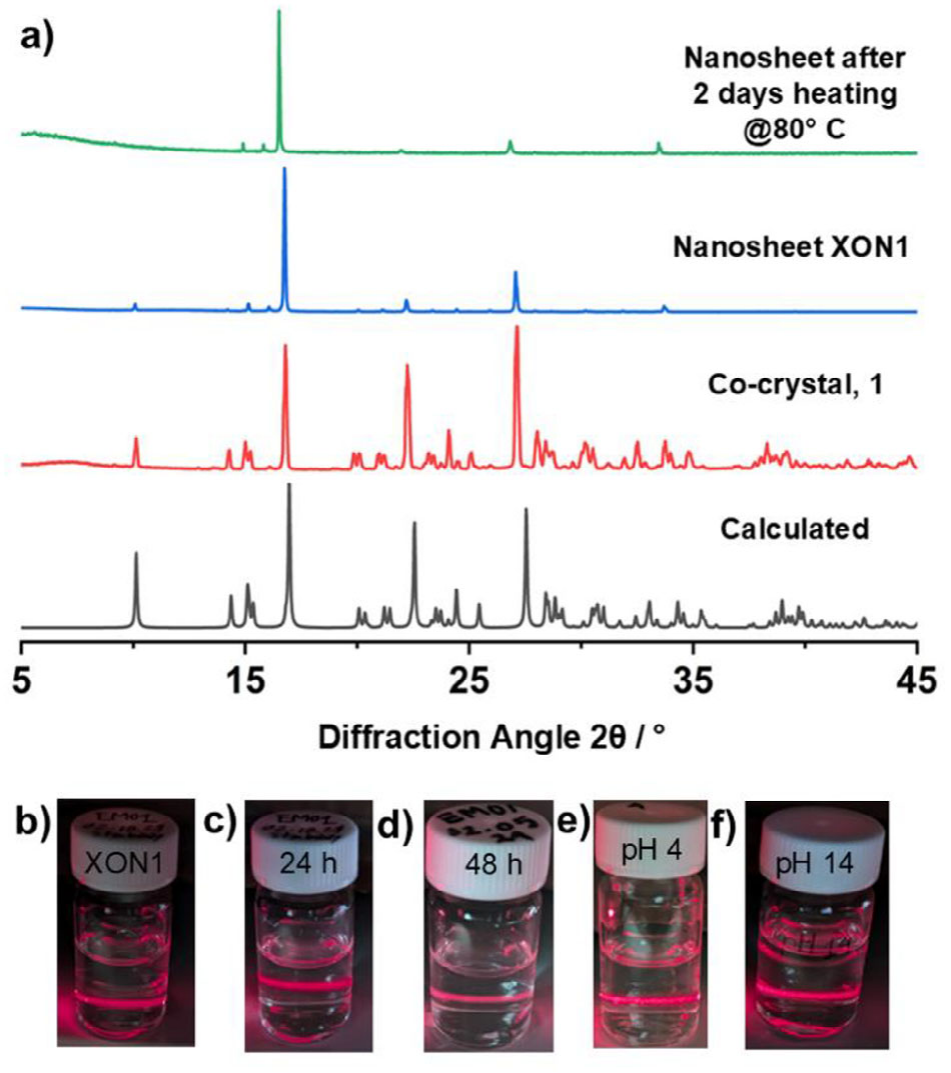
a) Powder X‐ray diffraction patterns for **1**: the calculated, as‐synthesized co‐crystal, nanosheet, and nanosheets after heating for 2 days at 80 °C. Tyndall scattering of nanosheet: b) as‐prepared, c) after 24 h of heating, d) after 48 h of heating, e,f) after acid and base treatment.

The stability of **XON1** was investigated further under a range of other conditions. **XON1** suspension maintained colloidal stability in water for up to seven days at room temperature, after which gradual dissolution was observed. Nanosheet suspensions were treated with different pH solutions of HCl_(aq)_ or NaOH_(aq)_, and their Tyndall scattering was monitored. **XON1** nanosheets showed no changes in Tyndall scattering or PXRD in the pH range 4–14 over several hours (**Figure** **3**e,f; Figures S42 and S43, Supporting Information), while partial dissolution was observed below pH 4, presumably due to disruption of halogen bonding caused by protonation of pyridyl groups.^[^ ^82^ ^]^ **XON1** continued to show Tyndall scattering following heating in water at 80 °C for 2 days (Figure 3d), with no changes in phase observed by PXRD (Figure 3a; Figure S40, Supporting Information). These results highlight the remarkable stability of these ultrathin XONs.

Samples of **2** were also exfoliated in water using the same sonication procedure to test their stability, and strong Tyndall scattering was observed. PXRD analysis of this partially exfoliated material matched the calculated patterns expected for **2** (Figures S31 and S32, Supporting Information), indicating that **2** has some stability in water at high concentrations. Attempts to isolate and fully characterize nanosheets of **2** under a variety of conditions were unsuccessful. Following the standard centrifugation step used to isolate **XON1** nanosheets, UV–Vis spectra of the resulting suspensions were found to closely match those of the dissolved **bpe** starting material (Figure S39, Supporting Information). PXRD patterns of material drop‐cast from suspension were dominated by **bpe‐H_2_O** (Figure S31, Supporting Information), with large needle‐like crystals observed to grow when the material was dried slowly (Figure S22, Supporting Information). **F_4_DBrB** was found to be highly insoluble in water and formed fine precipitates, with limited diffraction observed by PXRD (Figure S31, Supporting Information). AFM samples of **2** included a range of particle sizes and shapes, including some just a few nm thick (Figure S27, Supporting Information). These morphologies were found to match those produced in control experiments in which starting materials were exfoliated under the same conditions as for **1** (Figures S24 and S38, Supporting Information). TEM images of suspensions air‐dried on grids showed the presence of needle‐shaped materials rather than nanosheets (Figure S30, Supporting Information). These results indicate that, under the dilute conditions needed to isolate nanosheets, **2** disassociates into dissolved **bpe** and fine precipitates of **F_4_DBrB**.

These results highlight that the bromo‐nanosheets are less stable than the iodo‐versions, consistent with the expected weaker halogen bonding. This is borne out in other experimental data, such as the melting points (**1**: 236–240 °C; **2**: 130–135 °C).^[^ ^70^ ^]^ Competitive co‐crystallization experiments between acceptor **bpe** and mixtures of **F_4_DIB** and **F_4_DBrB** favored the formation of **1**, consistent with a stronger N···I interaction in **1** than the N···Br interaction in **2**.^[^ ^70^ ^]^ These differences were quantified with DFT calculations, with the binding energy for a single halogen‐bonded dimer calculated to be −8.8 kcal mol^−1^ for the iodo‐system and −5.3 kcal mol^−1^ for the bromo‐system, and the XB‐π nanosheet of the bromo‐system having a binding energy of −31.2 kcal mol^−1^ compared to −47.0 kcal mol^−1^ for **XON1**. DFT calculations also show that bromine‐based nanosheets have weaker binding energy in all directions at −22.6 and −32.3 kcal mol^−1^ for XB‐HB and HB‐π, respectively, which would be expected to result in less effective exfoliation.

## 3 Conclusion

Halogen bonds are an important class of intermolecular interactions that have been used to form a wide range of supramolecular architectures. Here, we report the first examples of their use to prepare free‐standing nanosheets formed by liquid exfoliation of molecular crystals. Two co‐crystals formed using iodo‐ or bromo‐containing halogen bond donors were exfoliated into a range of solvents by using an ultrasound bath. The iodo‐material produced micron‐sized nanosheets in water, approaching monolayer thickness with aspect ratios up to 500, and showed remarkable stability across a wide pH and temperature range. PXRD and DFT analysis of the nanosheets determined that weak hydrogen bonds between layers are broken during exfoliation in water, resulting in nanosheets held together by halogen bonds in one direction and *π*–*π* stacking interactions in a second.

This first study already highlights important design principles for creating stable XONs, such as using stronger iodo‐ rather than bromo‐halogen bond donors in combination with fluorinated halogen bond acceptors. Solubility is key to determining whether or not nanosheets can be formed in a particular solvent. The relative hydrophobicity of halogen bonds^[^ ^33^ ^]^ makes water an ideal choice of solvent for exfoliation, as the halogen bonds remain stable even within an ultrathin nanosheet layer in direct contact with solvent molecules, as well as acids and bases. The orthogonal properties of halogen bonds compared to other supramolecular interactions, such as hydrogen bonds and π–π stacking interactions, open up distinct opportunities for XONs as a new class of materials.

The conversion of layered inorganic materials into nanosheets has been revolutionary^[^ ^9^ ^]^ for a wide range of applications, which take advantage of their high surface areas and novel properties that arise from their nanoscopic dimensions. Molecular crystals are an even more numerous, diverse, and tunable class of materials with equal potential, yet only a handful of free‐standing nanosheets based on relatively strong hydrogen bonds have so far been reported because of the perception that strong in‐layer interactions are required to survive the high shear forces associated with liquid exfoliation. This work, therefore, highlights a significant new frontier in understanding the liquid exfoliation of layered materials in general and raises the question, “*How weak can we go?”* with in‐layer interactions within free‐standing nanosheets.

## Conflict of Interest

The authors declare no conflict of interest.

## Supporting information



Supporting Information

## Data Availability

The data that support the findings of this study are available in the supplementary material of this article.
