# Supplementary material for: Free‐Standing Halogen‐Bonded Nanosheets Formed by Ultrasonic Liquid Exfoliation
Source: Adv Sci (Weinh). 2025 Oct 29;13(3):e07800. doi: 10.1002/advs.202507800 (PMC12806269; doi:10.1002/advs.202507800)
Supplement: Supplementary file 1 — Supporting Information [file ADVS-13-e07800-s001.pdf]

Supporting Information

Free-standing Halogen-Bonded Nanosheets Formed by Ultrasonic Liquid Exfoliation

Prioti Choudhury Purba,[a] Elisa Marelli,[b] Thomas M. Roseveare,[a] Joshua Nicks,[a] Lee Brammer,[a] Nicholas G. White,[c] Natalia Martsinovich,[a] Giuseppe Resnati,[b] Pierangelo Metrangolo[b] and Jonathan A Foster*[a]

aSchool of Mathematical and Physical Sciences, University of Sheffield, Sheffield, UK. E-mail: jona.foster@sheffield.ac.uk

bDepartment of Chemistry, Materials, and Chemical Engineering "Giulio Natta", Politecnico di Milano, Via L. Mancinelli 7, 20131 Milan, Italy.

cResearch School of Chemistry, The Australian National University, Canberra, ACT 2601, Australia.

Contents

1. General considerations 1
2. Synthesis and characterisation of layered co-crystals 3
3. Preparation and characterisation of nanosheets 7
4. Stability test for nanosheets 17
5. Binding energy calculation 24
6. References 26

1. General considerations

1,2-Bis(4-pyridyl)ethylene, 1,4-diiodotetrafluorobenzene, and 1,4-dibromotetrafluorobenzene were purchased from Sigma Aldrich and used as received. All commercially available solvents were used as purchased without further purification.

X-Ray powder diffraction patterns were collected using a Bruker_AXS D8 Advance powder diffractometer equipped with a CuK_α source (λ=1.5418 Å). The instrument was fitted with an energy dispersive LYNXEYE position sensitive detector. Measurements were carried out in the 2θ range of 4 - 50° using a fixed goniometer stage in reflection geometry with a rotating flat zero-background silicon plate or in transmission geometry with packed 0.5 or 0.7 mm borosilicate capillaries.

X-Ray Powder diffraction data were analysed by Pawley refinement methods,[1] using TOPAS in conjunction with jEdit.[2,3] Assessments of the fit between the experimentally observed and calculated indices (R_{wp} and $R_{wp'}$) are defined by the equations below (equations **SE1** and **SE2**).

$$Rwp = \sqrt{\frac{\Sigma[w(Iobs - Icalc)^2]}{\Sigma[wIobs^2]}}$$

Supplementary Equation SE1. Index R_{wp} used in powder diffraction fitting. I_{obs}= observed intensity, I_{calc} = Calculated intensity, w= weighting factor.

$$Rwp' = \sqrt{\frac{\Sigma[w(Iobs-Icalc)^2]}{\Sigma[w(Iobs-bkgr)^2]}}$$

Supplementary Equation SE2. Index $R_{wp'}$ used in powder diffraction fitting. Bkgr = background terms.

Single-crystal diffraction experiments were performed using a Rigaku XtalLAB Synergy-R CuK_α rotating-anode X-ray diffractometer. Data were recorded using a HyPix-Arc 100˚ detector with temperature being maintained using an Oxford Cryosystems Cryostream 700 plus device series. Data were processed using CrysAlis Pro[4] with an empirical absorption correction using a spherical harmonic model being applied. Structure solution and refinement was performed in Olex2[5] using the SHELX suite of programs.[6] All non-hydrogen atom positions were modelled with anisotropic thermal displacement parameters. Hydrogen atoms were placed in calculated positions, refined with idealised geometries and assigned to fixed isotropic displacement parameters. Details of the structure solutions are outlined below (Tables **S1** and **S2**).

FT-IR spectra were recorded using a Perkin Elmer Spectrum 100 FT-IR spectrophotometer, equipped with a SenseIR diamond ATR module. Samples were analysed without further preparation, and spectra were obtained in reflectance mode, using 12 scans with a spectral resolution of 1 cm^{-1}.

Elemental analyses were performed by the microanalytical service at the Department of Chemistry, University of Sheffield using a Vario MICRO Cube in an atmosphere of pure O_2 during the combustion sequence of the analysis, the remainder using helium as a carrier gas in the absence of oxygen. Elemental contents are determined to a tolerance of ± 0.3 %.

Samples were prepared for scanning electron microscopy (SEM) by loading crystalline sample powders onto carbon sticky tape placed on aluminium SEM sample stubs, coated with approximately 20 nm gold using an Edwards S150B gold sputter coater and loaded into a TESCAN VEGA3 LMU SEM instrument. Nanosheet suspensions were freeze-dried before SEM sample preparation. All SEM images reported in this work were collected with a secondary electron detector.

Transmission electron microscopy (TEM) and selected-area electron diffraction (SAED) analysis for **XON1** were performed using Philips CM200 field-emission microscope operated at 200 kV in bright field mode (for TEM) with Omega-type Zero-loss energy filter. TEM imaging of suspension of **2** was performed using a FEI Tecnai Biotwin G2 equipment with a Gatan Orius 1000B digital camera operated at 80 kV in bright field mode. The samples were prepared by drop-casting 10 μL nanosheet suspension on a 200-mesh carbon film-coated grid and the sample was allowed to settle down for 1 min. The excess water was removed with filter paper after 1 min to prevent aggregation effects promoted by drying.

Atomic force microscopy (AFM) images were recorded using a Bruker Multimode 5 Atomic Force Microscope, operating in soft-tapping mode in air under standard ambient temperature and pressure, and fitted with Bruker OTESPA-R3 silicon cantilevers operated with a drive amplitude of ~18.70 mV and resonance frequency of ~236 kHz. Samples were prepared by drop-casting 10 μL of nanosheet suspension onto the centre of freshly cleaved mica sheets heated to 80 °C for **XON1** and 65 °C for suspension of **2** on a hot plate. These mica sheets were attached with adhesive to stainless steel, magnetic Agar scanning probe microscopy

specimen discs. All AFM images reported in this work were processed using Gwyddion software.[7]

Dynamic Light Scattering data were collected using a Malvern Zetasizer Nano Series particle size analyser, using a He-Ne laser at 633 nm, operating in backscatter mode (173°). Samples were placed in quartz cuvettes and equilibrated at 298 K for 60 s prior to analysis.

Ultraviolet-visible (UV-vis) absorption spectra were collected on a Cary 60 UV-vis instrument, using a 1 cm internal length quartz cuvette and Cary WinUV program. Scan software was used for absorption spectra recording under room temperature.

2. Synthesis and characterisation of layered co-crystals

Both the layered co-crystals, **1** and **2** were synthesised by following a previously reported procedure.[8]

Synthesis of 1: Equimolar solution of 1,2-bis(4-pyridyl)ethylene (227.0 mg) and 1,4-diiodotetrafluorobenzene (500.0 mg) in chloroform (5.0 mL) were mixed in a clear borosilicate glass vial at room temperature and in a few minutes colourless co-crystals of **1** were obtained. Yield: 71%. FT-IR (inter alia): 3058, 3035, 1598, 1450, 1199, 970, 934, 822, 749, 543 cm^{-1} (Figure S1). Elemental analysis: calcd (%) for $C_{18}H_{10}N_2F_4I_2$: C, 37.0; N, 4.8; H, 1.7; found (%): C, 40.4; N, 5.0; H, 1.5. Confirmation of phase purity of the isolated solid was obtained by PXRD (Figures S3 and S4).

Synthesis of 2: Equimolar amount of 1,2-bis(4-pyridyl)ethylene (237.0 mg) and 1,4-dibromotetrafluorobenzene (400.0 mg) were dissolved in chloroform (6.0 mL) separately and mixed in a vial of clear borosilicate glass. Chloroform was allowed to evaporate at room temperature and after two days, cocrystal **2** was obtained as colourless crystals. Yield: 73%. FT-IR (inter alia): 3058, 3032, 1597, 1474, 1411, 995, 952, 820, 784, 550 cm^{-1} (Figure S2). Elemental analysis calcd (%) for $C_{18}H_{10}Br_2F_4N_2$: C 44.1, H 2.0, N 5.7; found (%): C, 49.3; H, 1.9; N, 6.3. Confirmation of phase purity of the isolated solid was obtained by PXRD (Figures S5 and S6).

Infrared Spectroscopic Data

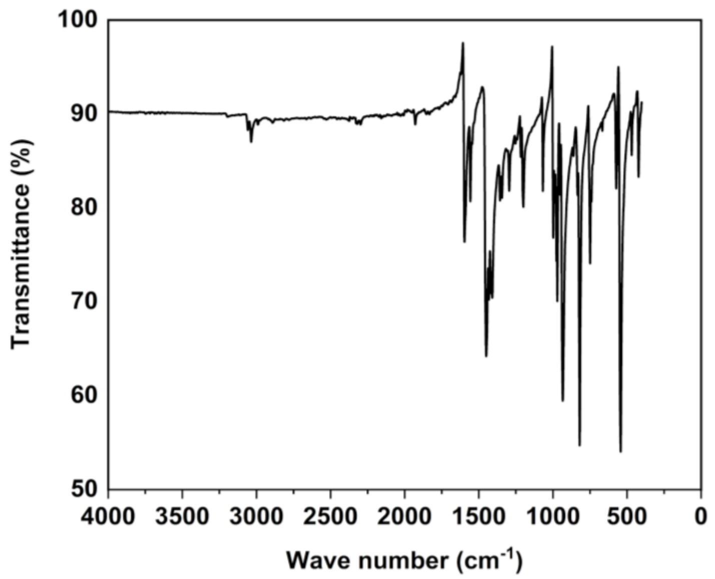

Figure S1. IR spectrum of co-crystal **1**.

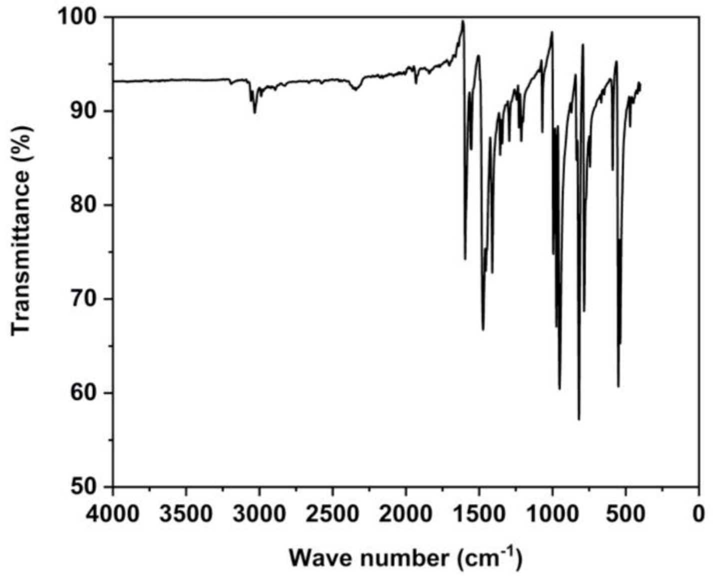

Figure S2. IR spectrum of co-crystal **2**.

Powder X-Ray Diffraction

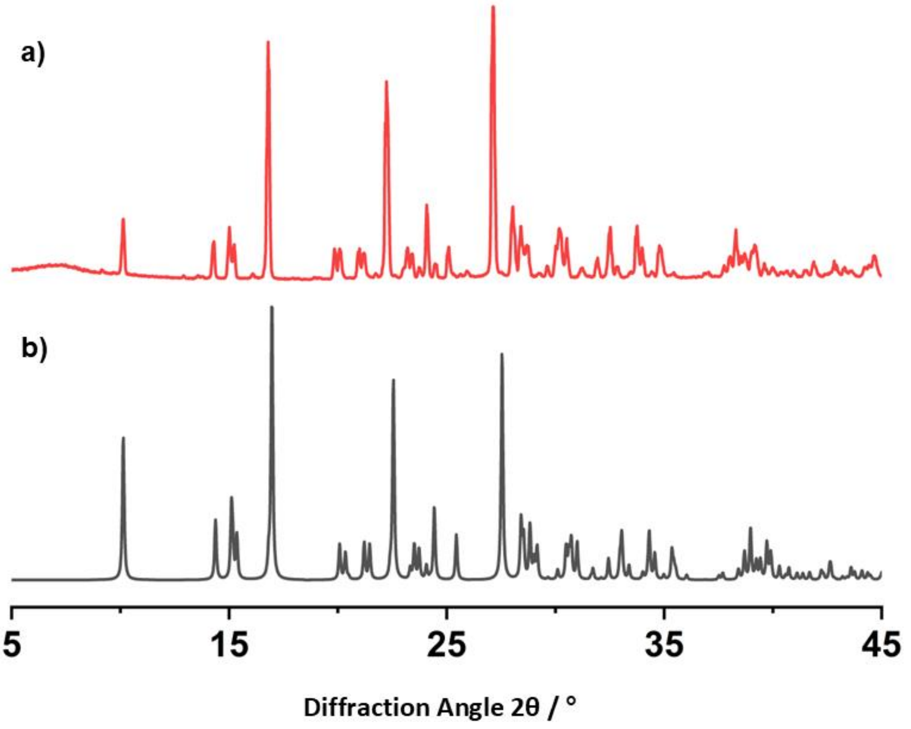

Figure S3. PXRD pattern of a) as-synthesised **1** and b) calculated from room temperature single-crystal structure of **1**.[8b]

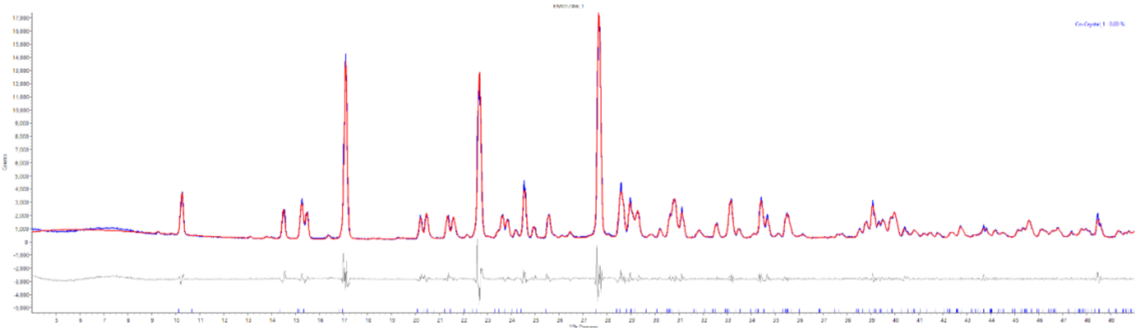

Figure S4. Pawley refinement of the PXRD pattern of as synthesised **1**, observed (blue), calculated (red) and difference plot [I_{obs}-I_{calc}] (grey) (2θ range 4-50 ˚). Refinement details **1**: Space group: *P-1*, Volume = 454.84(7) Å^3, a = 6.2853(6) Å, b = 8.4651(8) Å, c = 9.2594(8) Å, α = 83.522(2) ˚, β = 70.716(3) ˚, λ = 78.337(2) ˚, R_{wp} = 0.0988, $R_{wp'}$ = 0.1447, parameters 185 (9 background, 2 radiation contamination, 1 zero error, 5 peak profile, 6 lattice parameters and 164 reflections).

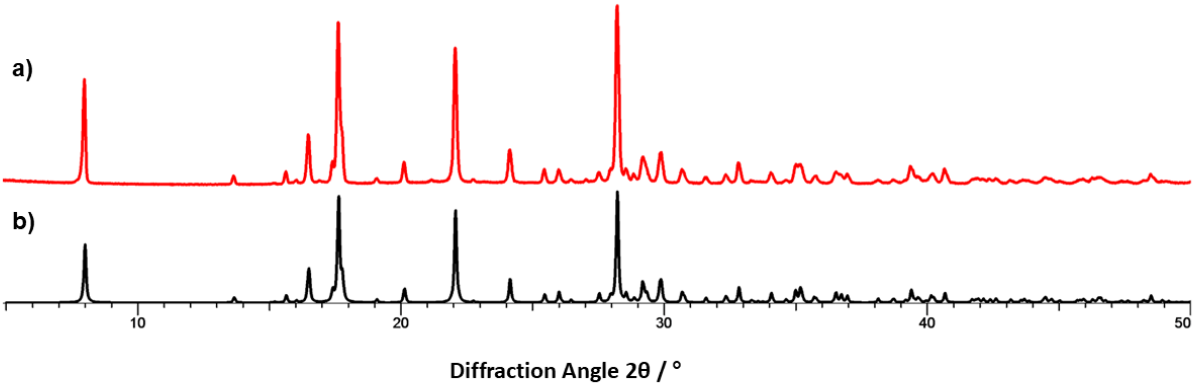

Figure S5. PXRD pattern of a) as-synthesised **2** (recorded in transmission geometry with packed borosilicate capillaries) and b) calculated from room temperature single-crystal data (CSD identifier: IKUHUR01) of **2**.[8b]

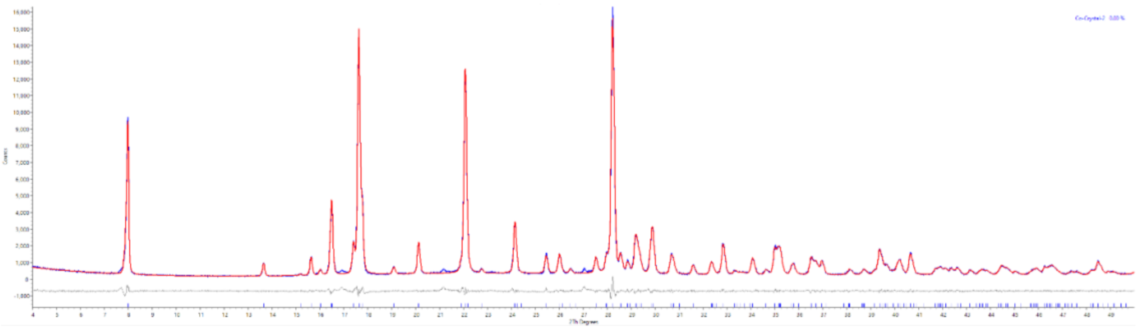

Figure S6. Pawley fit of the PXRD pattern of as synthesised **2**, observed (blue), calculated (red) and difference plot [I_{obs}-I_{calc}] (grey) (2θ range 4-50 ˚). Refinement details **2**: Space group: *P-1*, Volume = 439.12(2) Å^3, a = 6.1105(2) Å, b = 7.0182(2) Å, c = 11.1190(3) Å, α = 67.930(1) ˚, β = 86.314(2) ˚, λ = 83.730(1) ˚, R_{wp} = 0.0624, $R_{wp'}$ = 0.0934, 172 parameters (9 background, 1 zero error, 5 peak profile, 6 lattice parameters and 151 reflections).

X-Ray Crystal Structure of 2

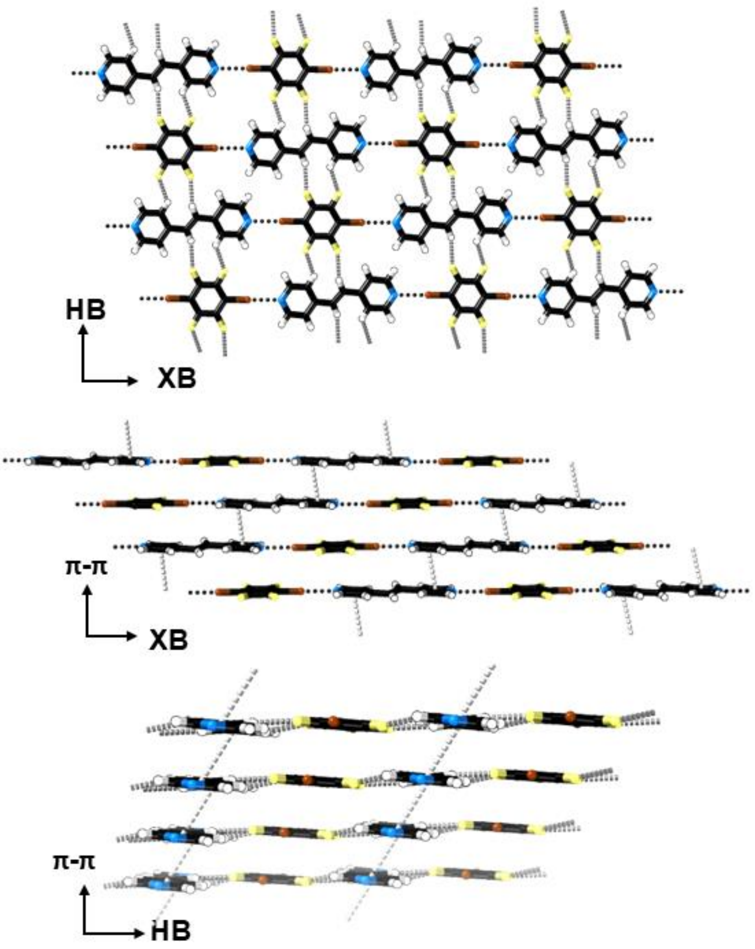

Figure S7. X-Ray crystal structure of **2**,[8b] showing different combinations of halogen bonding (XB), hydrogen bonding (HB) and π - π stacking interactions. Colour codes: C (grey), H (white), N (blue), F (yellow) and Br (brown). Halogen and hydrogen bonds shorter than the sum of the van der Waals radii and aromatic contacts with centroid•••centroid distances less than 4 Å are shown with dotted lines.

SEM Imaging of 1 and 2

SEM images of as-synthesised **1** and **2** are shown in Figures S8 and S9 at multiple magnifications.

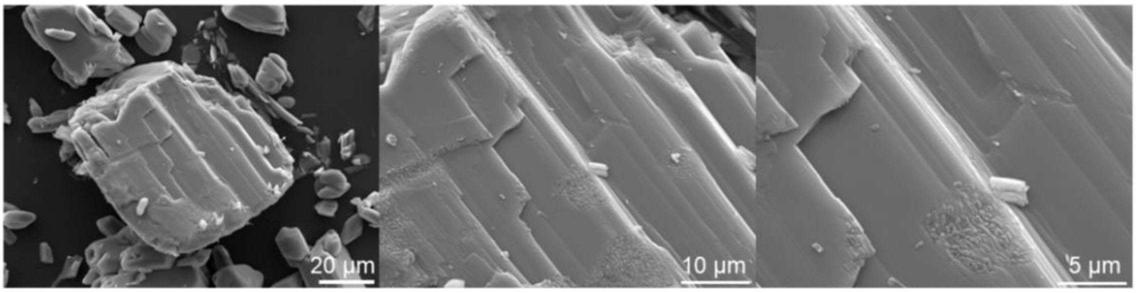

Figure S8. SEM images at 20 µm, 10 µm and 5 µm scales, indicating the layered morphology of **1**.

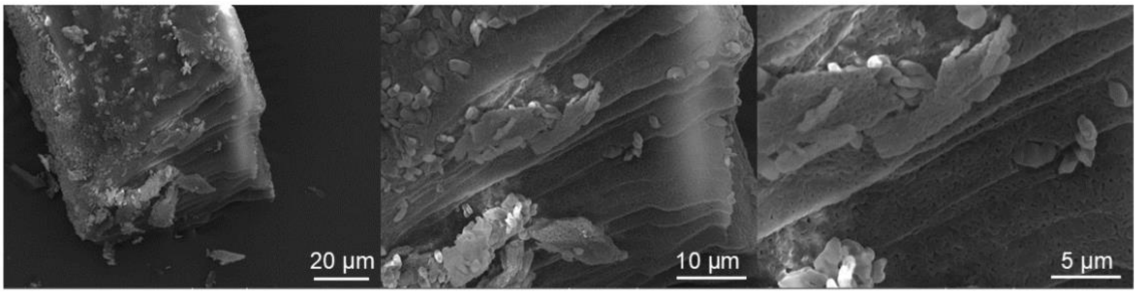

Figure S9. SEM images at 20 µm, 10 µm and 5 µm scales, indicating the layered morphology of **2**.

3. Preparation and Characterisation of Nanosheets

Ultrasonic Exfoliation

Samples (5 mg) of **1** and **2** were suspended in water (6 mL) and vortexed for 20 s, followed by sonication for 6 h using a Fisher Elmasonic P 30H ultrasonic bath operating at 80 kHz and 100% power (320 W). Samples were rotated using an overhead stirrer to ensure even exposure and prevent the occurrence of “hot spots”, and the bath was fitted with a water coil to maintain temperature at approximately 18–20 °C. After sonication, samples were transferred into polypropylene centrifuge tubes and centrifuged for 10 minutes at 600 rpm followed by another 10 minutes of centrifugation at 800 rpm. Centrifugation of the suspension allows unexfoliated material to settle down at the bottom and leaves nanosheets in suspension.[9]

Nanosheet Characterisation

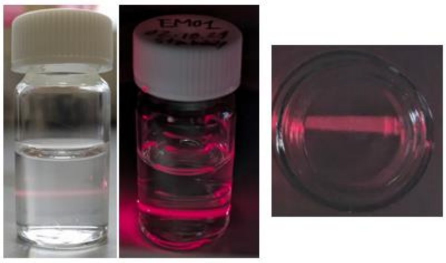

Figure S10. Tyndall scattering effects exhibited by as-prepared suspensions of **1** in water under day light and dark conditions (side and top view).

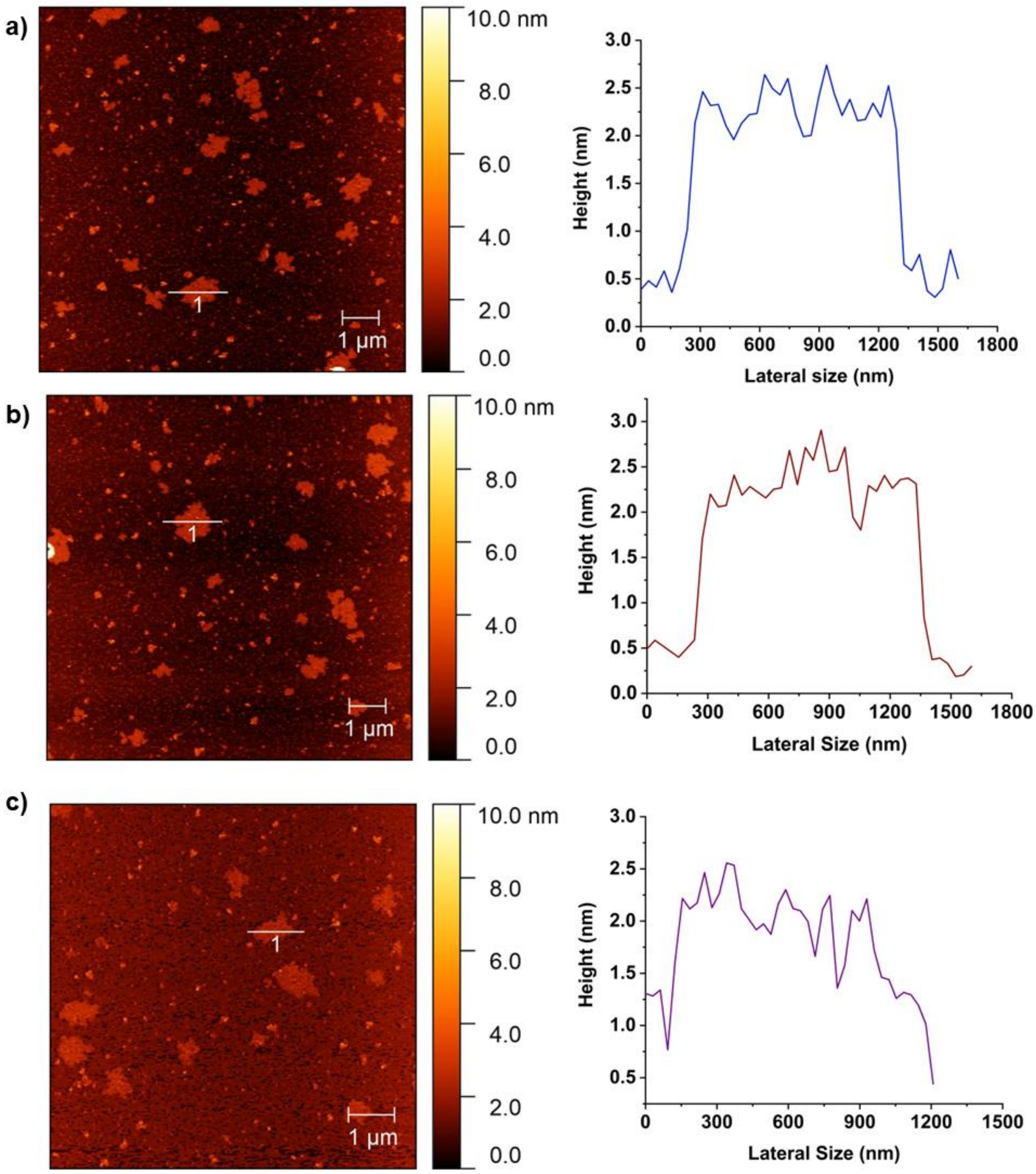

Figure S11. AFM topographical images of **XON1** showing uniform thickness of nanosheets from different areas of the sample.

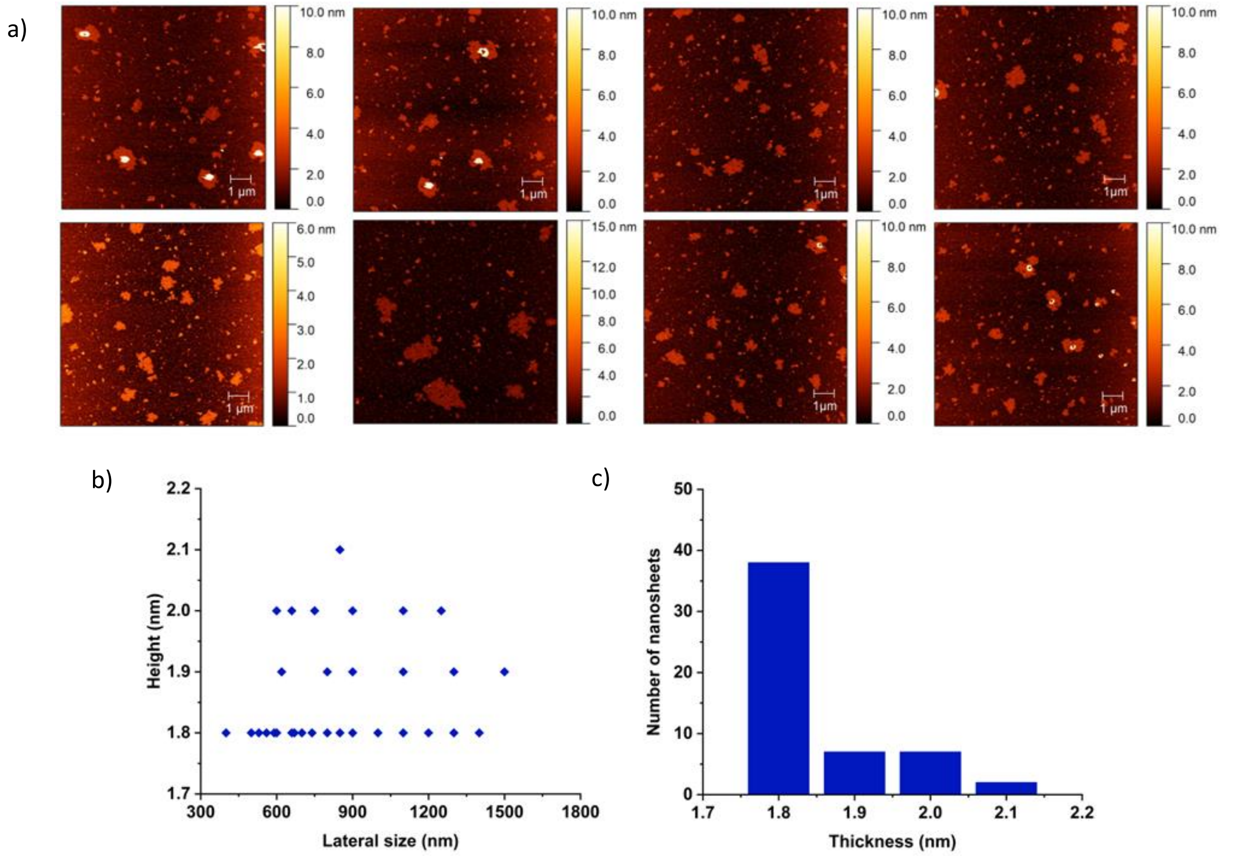

Figure S12. a) AFM topographical images of aqueous suspensions of **XON1** deposited onto Micah via a hot-drop method. b) Scatter plot and c) thickness histogram made by drawing line profiles across the wides part of separate nanosheets.

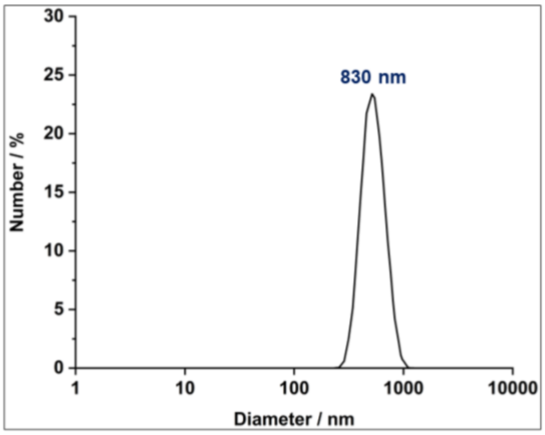

Figure S13. Dynamic Light Scattering (DLS) number plot for suspension of **1** in water.

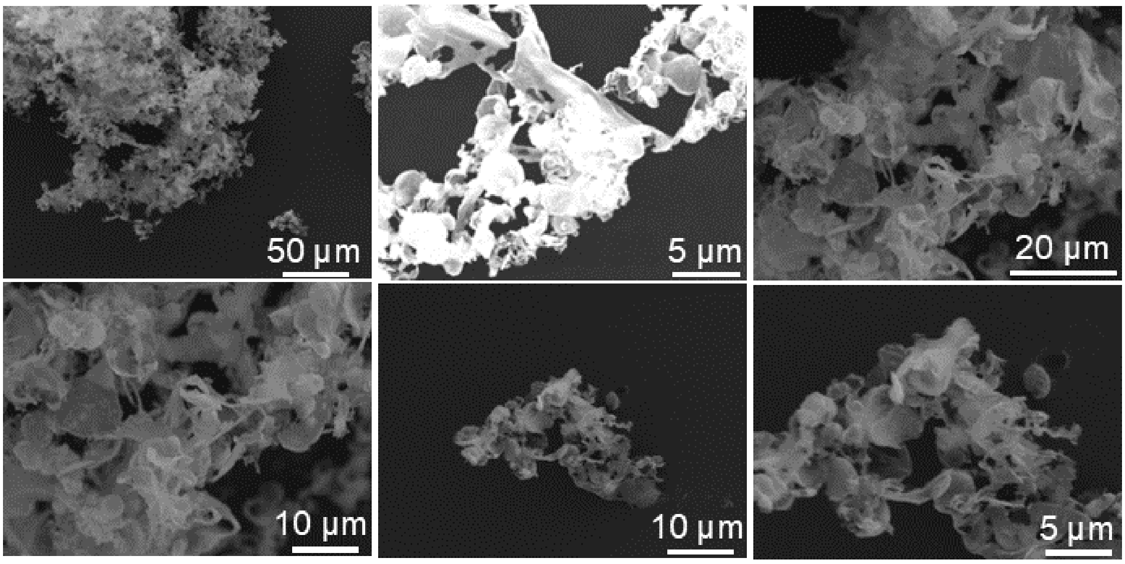

Figure S14. SEM images of **XON1** nanosheets (freeze-dried) at multiple magnifications.

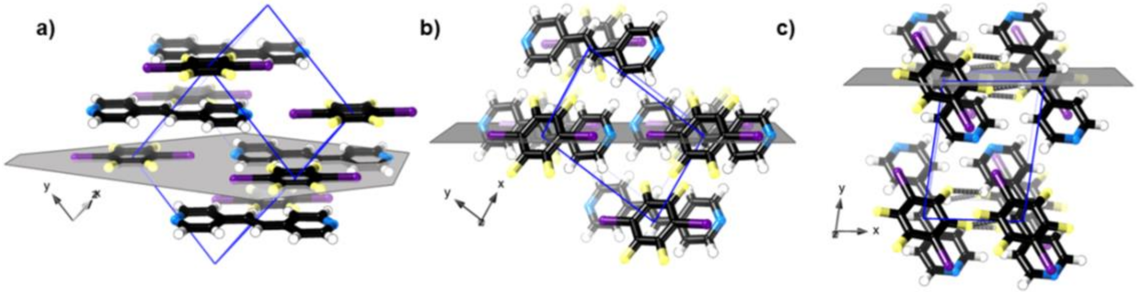

Figure S15. Crystal structures of **1**[8b] showing (a) (1 2 2) plane (XB-HB) containing 1D halogen bonded chains which are hydrogen bonded in the second dimension; (b) (1 1 0) plane (XB-π) containing 1D halogen bonded chains which are π – π stacked in the second dimension; (c) (0 1 0) plane (HB-π) containing hydrogen bonded chains which are π – π stacked in the second dimension. The unit cell is shown in blue. Colour codes: C (black), H (white), N (blue), F (yellow) and I (purple).

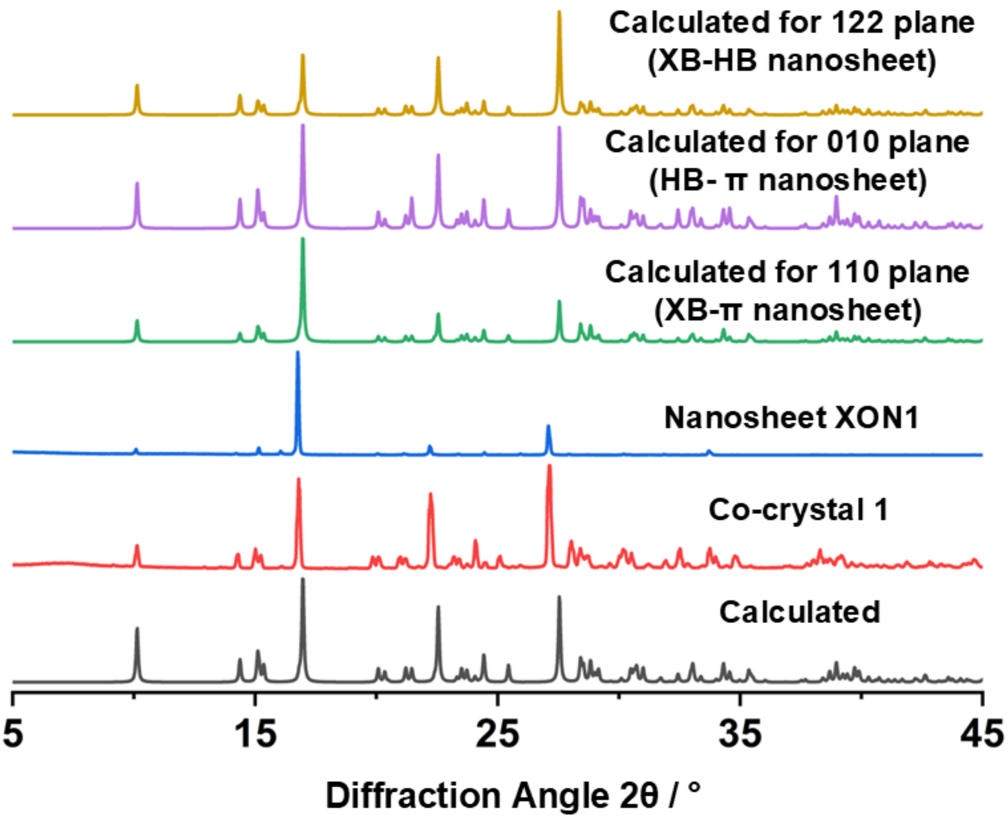

Figure S16. Powder X-ray diffraction patterns for **1**: the calculated from room temperature single-crystal structure, as synthesised co-crystal, nanosheet **XON1**, calculated preferred-orientation pattern for (1 1 0) plane (XB-π nanosheet), (0 1 0) plane (HB-π nanosheet) and (1 2 2) plane (XB-HB nanosheet). For preferred-orientation pattern calculation, a Dollase parameter[10] of 0.8 was used.

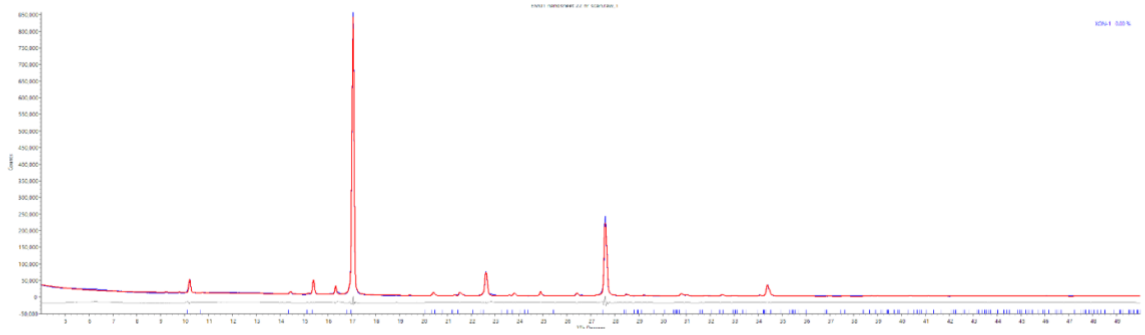

Figure S17. Pawley fit of PXRD pattern for Nanosheet **XON1**, observed (blue), calculated (red) and difference plot [I_{obs}-I_{calc}] (grey) (2θ range 4-50 ˚). Refinement details **1**: Space group: *P-1*, Volume = 455.2(1) Å^3, a = 6.287(1) Å, b = 8.468(1) Å, c = 9.2574(9) Å, α = 83.508(7) ˚, β = 70.745(8) ˚, λ = 78.401(9) ˚, R_{wp} = 0.0642, $R_{wp'}$ = 0.1200, 185 parameters (9 background, 2 radiation contamination, 1 zero error, 5 peak profile, 6 lattice parameters and 164 reflections).

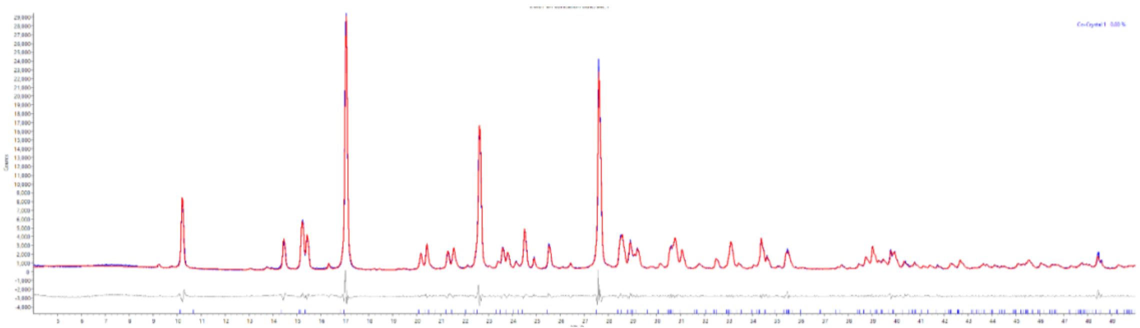

Figure S18. Pawley fit of PXRD pattern for partially exfoliated material **1** following 6 hours sonication, observed (blue), calculated (red) and difference plot [I_{obs}-I_{calc}] (grey) (2θ range 4-50 ˚). Refinement details **1**: Space group: *P-1*, Volume = 454.97(6) Å^3, a = 6.2855(4) Å, b = 8.4666(6) Å, c = 9.2602(6) Å, α = 83.506(2) ˚, β = 70.717(2) ˚, λ = 78.332(2) ˚, R_{wp} = 0.0778, $R_{wp'}$ = 0.1076, 185 parameters (9 background, 2 radiation contamination, 1 zero error, 5 peak profile, 6 lattice parameters and 164 reflections).

UV-Vis Experiments

UV-Vis absorption spectroscopy was used to quantify the mass concentration of **XON1**. The standard exfoliation and centrifugation protocols were applied to **bpe** and **F4DIB** as well as **XON1** and UV-Vis spectra recorded. **F4DIB** was found to only be sparingly soluble, with most material remaining at the bottom of the vial. **bpe** produced clear solutions with only very faint signs of Tyndal scattering so was assumed to have fully dissolved for the purpose of calculations. The concentration of **XON1** in suspension was estimated by measuring the mass loss from the partially exfoliated weighted sample. Serial dilution of the samples produced calibration curves from which extinction coefficients were measured for **bpe** (32885 $M^{-1}cm^{-1}$) and **XON1** (1539 $M^{-1}cm^{-1}$) (Figures S19 and S20) .

The concentration of **XON1** was estimated using extinction coefficient value. The concentration of the nanosheet suspension was calculated to be 0.08 mg mL^{-1}, corresponding to 9 wt% yield of the dispersed material remaining in suspension following centrifugation.

Samples of same **XON1** were then centrifuged at 6000 rpm for 20 minutes to remove suspended material and the supernatant measured by UV-Vis to allow the concentration of free **bpe** in solution to be determined. No absorbance at longer wavelengths was observed indicating successful removal of all nanosheets and the UV-Vis spectra of the supernatant matched that of **bpe** (Figure S21b). Based on the extinction coefficient for **bpe** at 299 nm, the concentration of dissolved **bpe** remaining in solution was calculated to be 0.00025 mg mL^{-1}, indicating negligible (0.09 %) dissolution of **XON1**.

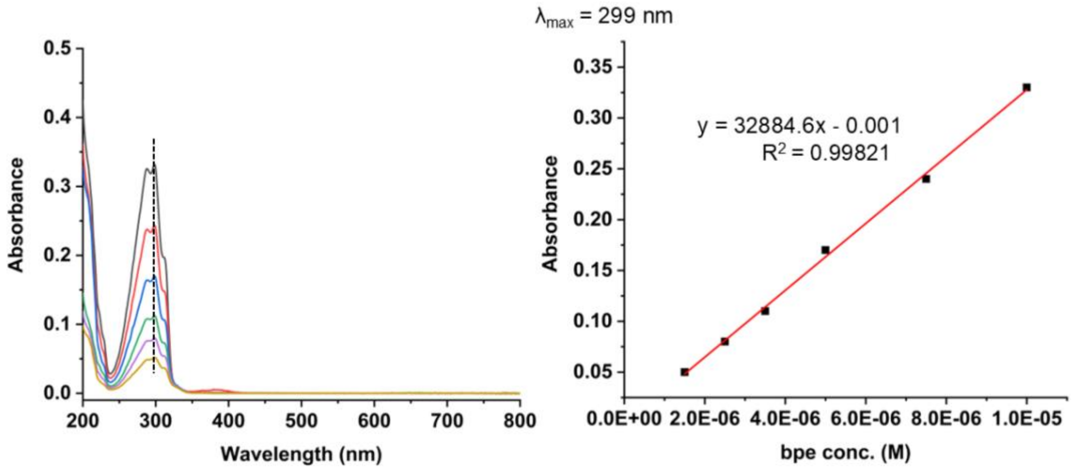

Figure S19. UV-vis stack plot and extinction coefficient calculation graph for **bpe** exfoliated in water.

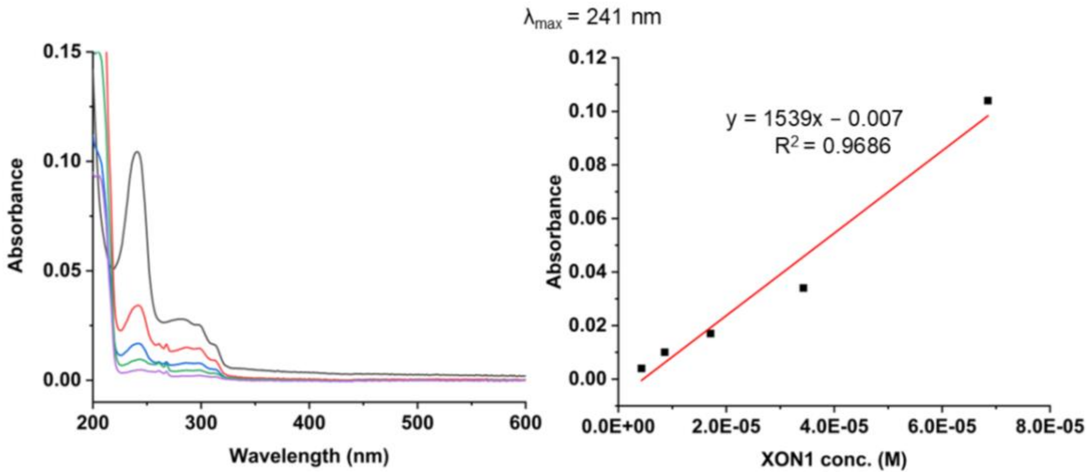

Figure S20. UV-vis stack plot and extinction coefficient calculation graph for **XON1** exfoliated in water.

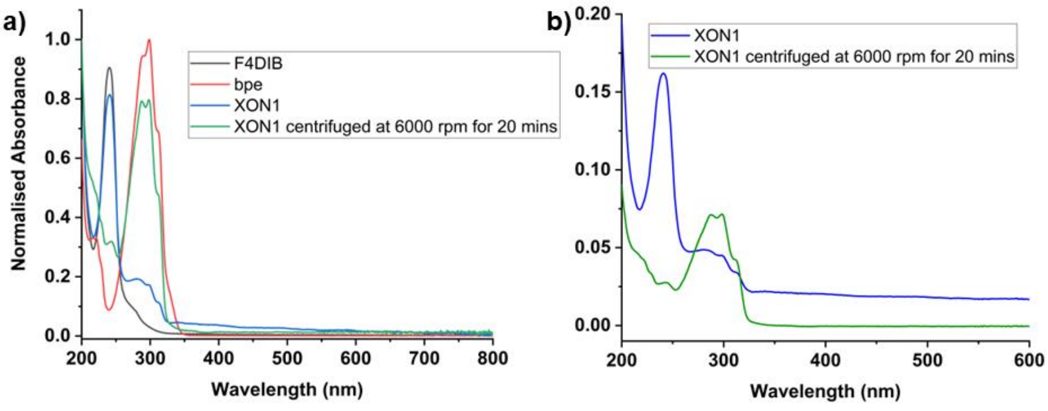

Figure S21. a) Normalised UV spectra of **bpe**, **F_4DIB**, **XON1** before and after centrifugation at 6000 rpm for 20 mins, b) UV-Vis stack plot of **XON1** suspension before and after centrifugation at 6000 rpm for 20 mins. Nanosheet concentration was calculated using this spectrum.

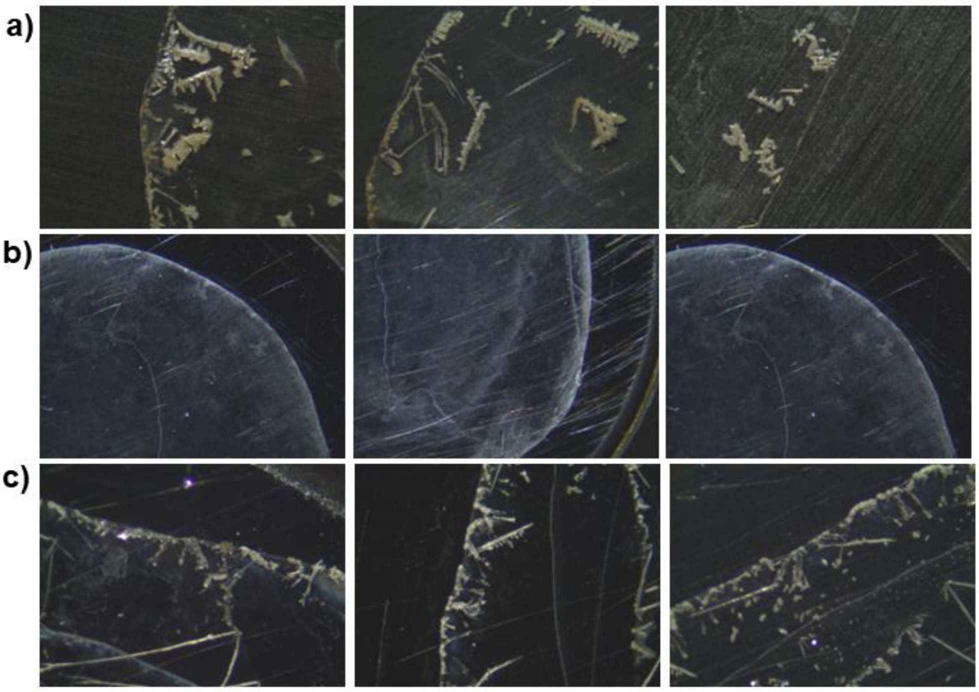

Figure S22. Images taken under the microscope showing a) **bpe**, b) **XON1** suspensions deposited on aluminium foil. c) Images of exfoliated **2** suspension deposited on zero-background flat plate.

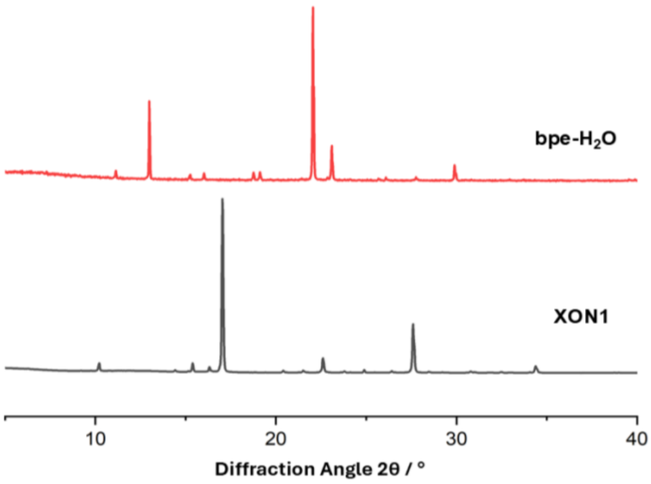

Figure S23. PXRD pattern of **XON1** (bottom) and **bpe-H_2O** when **bpe** suspension was deposited on a flat plate.

Control experiments were carried out in which starting materials were exfoliated under the same exfoliation conditions as for **1**and their AFM images (figure S24-25) were recorded to compare with the nanosheet **XON1**.

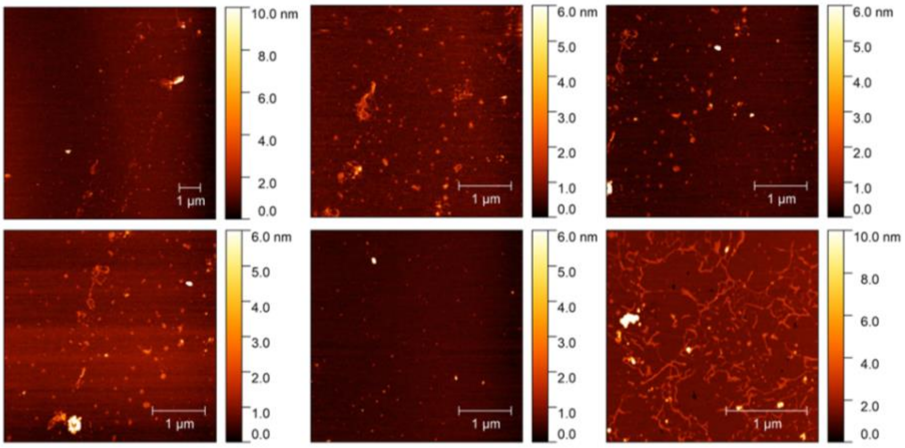

Figure S24. AFM images of the **bpe** suspension in water.

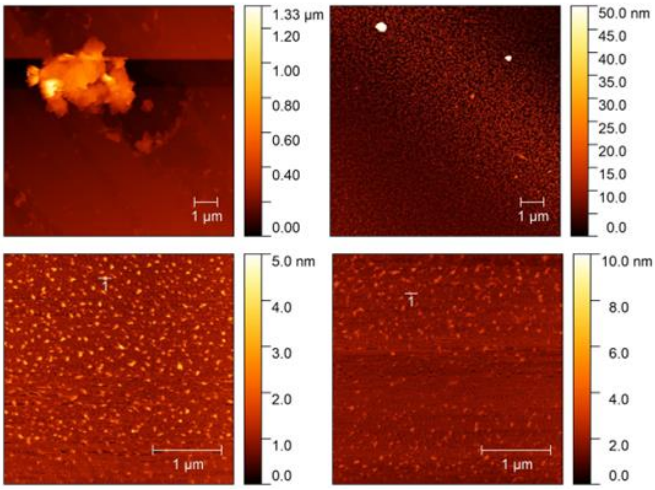

Figure S25. AFM images of the **F_4DIB** suspension in water.

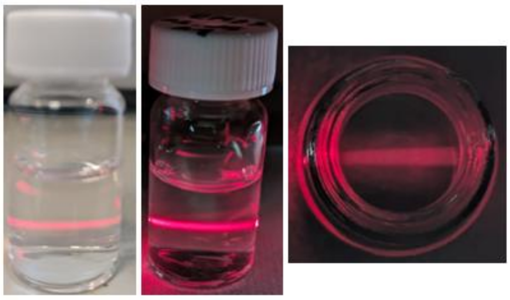

Figure S26. Tyndall scattering effects exhibited by as-prepared suspensions **2** in water under daylight and dark conditions (side and top view).

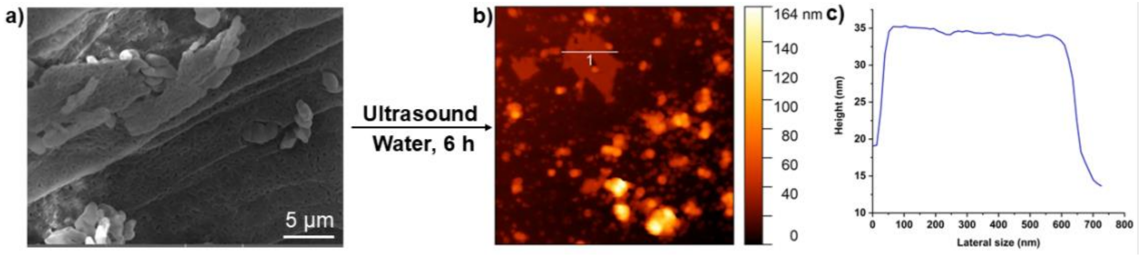

Figure S27. Ultrasonic liquid exfoliation of a) layered cocrystal, **2** in water medium to form b) nanosheet. C) height profile of the nanosheet.

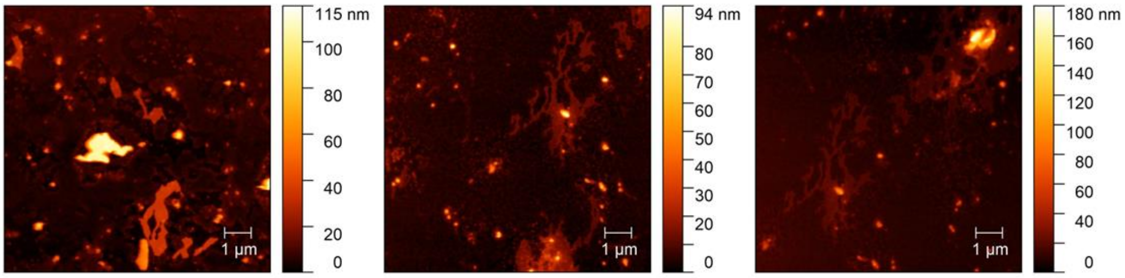

Figure S28. AFM topographical images of exfoliated **2** from different areas of the sample.

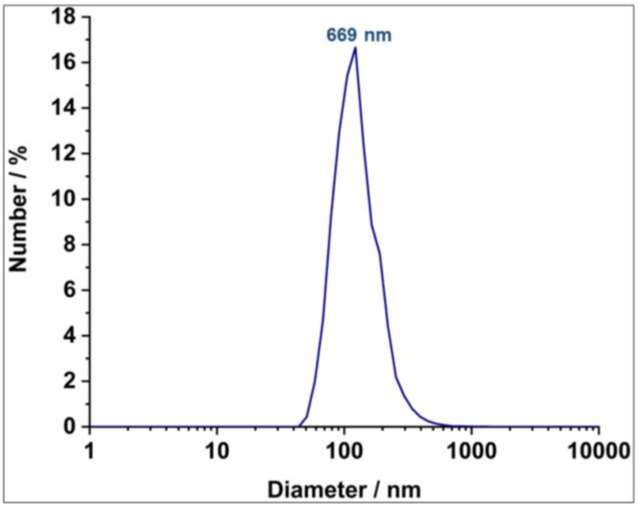

Figure S29. Dynamic Light Scattering (DLS) number plot for suspension of **2** in water.

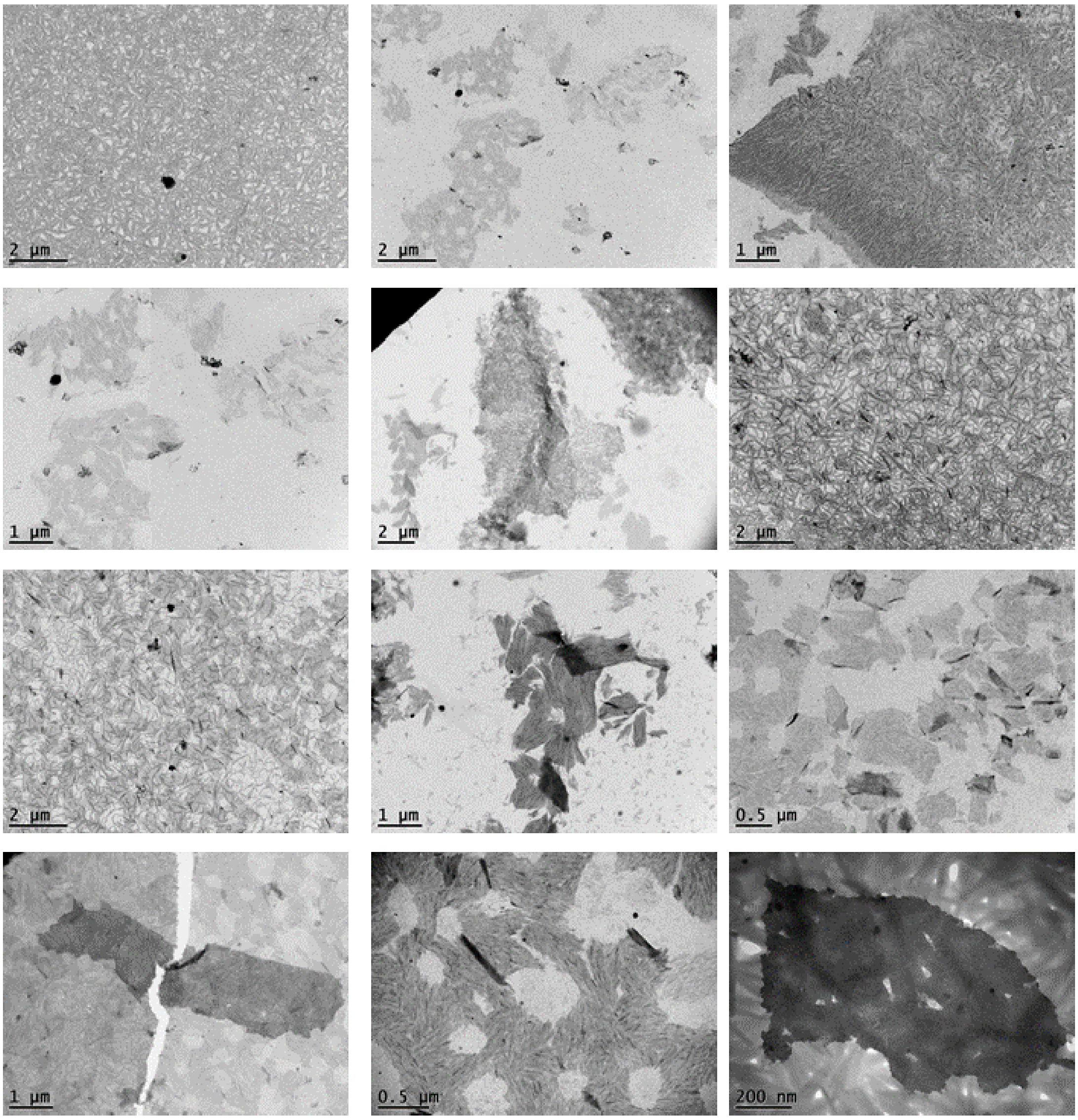

Figure S30. TEM images of the suspension of **2** from different parts of the sample.

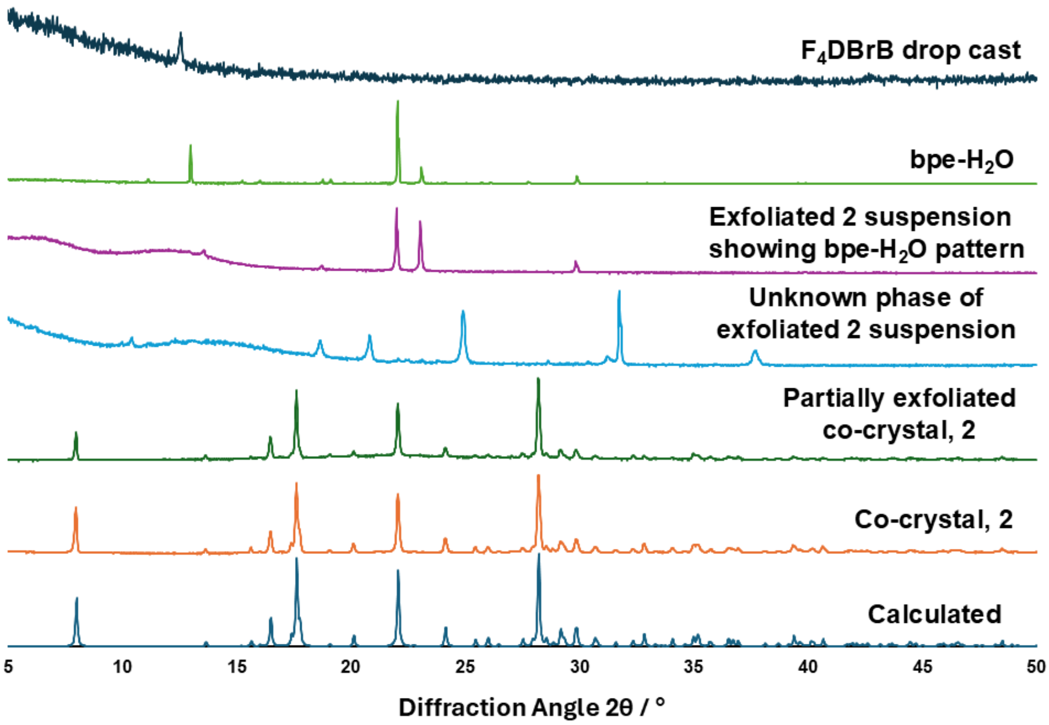

Figure S31. Powder X-ray diffraction patterns for **2** (top to bottom): calculated from room temperature single-crystal structures (CSD identifier: IKUHUR01)[8b] of **2**, as synthesised co-crystal, partially exfoliated co-crystal, unknown phase obtained by repeated drop casting of exfoliated suspension, drop cast of exfoliated suspension of **2** showing pattern of **bpe-H_2O**, **bpe-H_2O** and, **F4DIB** drop cast.

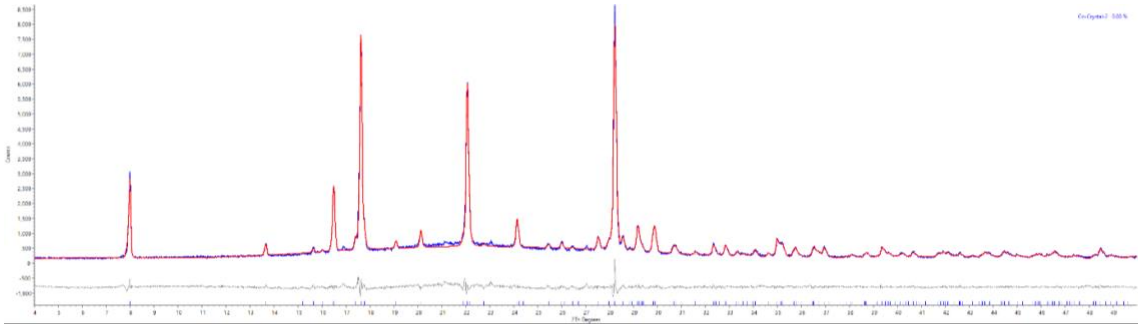

Figure S32. Pawley fit of partially exfoliated **2**, observed (blue), calculated (red) and difference plot [I_{obs}-I_{calc}] (grey) (2θ range 4-50 ˚). Refinement details **2** Space group: *P-1*, Volume = 439.32(4) Å^3, a = 6.1119(3) Å, b = 7.0201(3) Å, c = 11.1195(6) Å, α = 67.917(3) ˚, β = 86.308(4) ˚, λ = 83.744(3) ˚, R_{wp} = 0.0794, $R_{wp'}$ = 0.1714, 173 parameters (9 background, 1 zero error, 5 peak profile, 6 lattice parameters and 152 reflections).

PXRD data obtained from repeated drop cast of exfoliated suspension initially produced a crystalline material that could not be attributed to the desired co-crystal structure or any of the starting materials (Figure S31, unknown phase). When this sample holder was inspected using a microscope, there were a small number of single crystals present, which were expected to result from slow evaporation of water when preparing the drop-cast samples. X-Ray diffraction analysis of a single crystal revealed that this was 1,2-bis(4-pyridyl)ethylene hydrate (**bpe-**

H_2O). However, all further attempts to prepare nanosheets of **2** only produced XRPD patterns consistet with formation of bpe hydrate.

Summary details of the structure solution of **bpe-H_2O** are summarised in **Table S1**.

Table S1. Summary details of structure solution of **bpe-H_2O**, crystal isolated from repeated drop casting of suspension of **2** from H_2O

	**bpe-H_2O**<br>$C_{12}H_{12}N_2O$<br>200.24
Crystal habit	Plate
Crystal colour	Colourless
Crystal size (mm)	0.504 × 0.032 × 0.02
Crystal system	Orthorhombic
Space group, Z	*Pna2₁*, 4
a (Å)	9.4794(5)
b (Å)	14.7865(5)
c (Å)	7.7141(3)
α (°)	90
β (°)	90
γ (°)	90
V (Å^3)	1081.26(8)
Density (g cm^{-3})	1.23
Temperature (K)	298.13(10)
μ (mm^{-1})	0.642
Radiation (λ)	Cu Kα (λ = 1.54184)
2θ range (°)	11.088 to 155.802
Reflns. collected	10392
Independent reflns. (R_{int})	2125 (R_{int} = 0.0650)
Reflns. used in refinement, n	2125
LS parameters, p	139
Restraints, r	1
R1 (F)[a] I>2σ(I)	0.072
wR2 (F^2)[a], all data	0.2056
S(F^2)[a], all data	1.06
Flack Parameter	0.3(4)

[a]$R1(F) = \Sigma(|F_o| - |F_c|)/\Sigma|F_o|$; $wR2(F^2) = [\Sigma w(F_o^2 - F_c^2)^2/\Sigma wF_o^4]^{1/2}$; $S(F^2) = [\Sigma w(F_o^2 - F_c^2)^2/(n + r - p)]^{1/2}$

The dissolution of bromo-nanosheet and the drastic phase change of bromo-nanosheet (unknown phase) (Figure S31) upon exfoliation prompted thermal stability studies of the as-synthesised co-crystal, **2**. Firstly, a sample of **2** was soaked in water for 6 h (same as sonication time) and dried at room temperature. This treatment resulted in retention of desired cocrystal **2** (Figure S33).

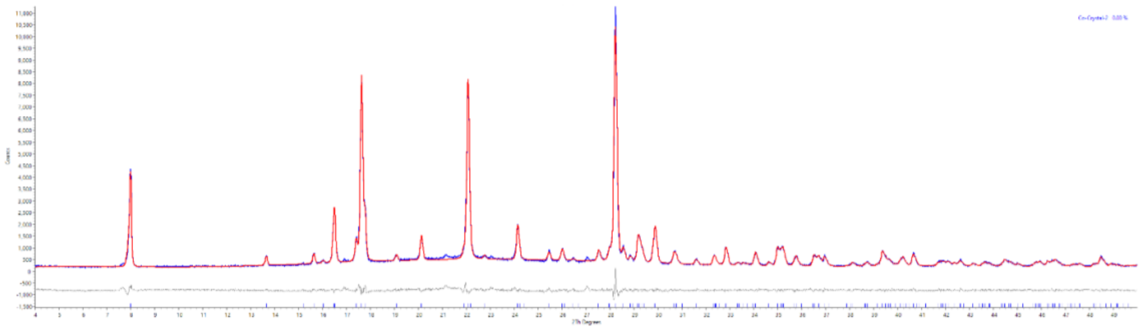

Figure S33. Pawley fit of PXRD pattern of **2** (sample soaked in water for 6 h and dried in air at room temperature), observed (blue), calculated (red) and difference plot [I_{obs}-I_{calc}] (grey) (2θ range 4-50 ˚). Refinement details of **2**: Space group: *P-1*, Volume = 439.42(3) Å^3, a = 6.1121(2) Å, b = 7.0200(2) Å, c = 11.1215(4) Å, α = 67.921(2) ˚, β = 86.309(2) ˚, λ = 83.736(2) ˚, R_{wp} = 0.0718, $R_{wp'}$ =0.1316, 173 parameters (9 background, 1 zero error, 5 peak profile, 6 lattice parameters and 152 reflections).

Secondly, samples of co-crystal **2** were treated in two different ways: 1) *sonicated in water for 6 h and then heated at 80 ˚C for a total of 5 minutes* and 2) *soaked in water for 6 h and then heated at 80 ˚C for a total of 5 minutes*. This short heating regime showed minimal change to the air dried and as-synthesised materials.

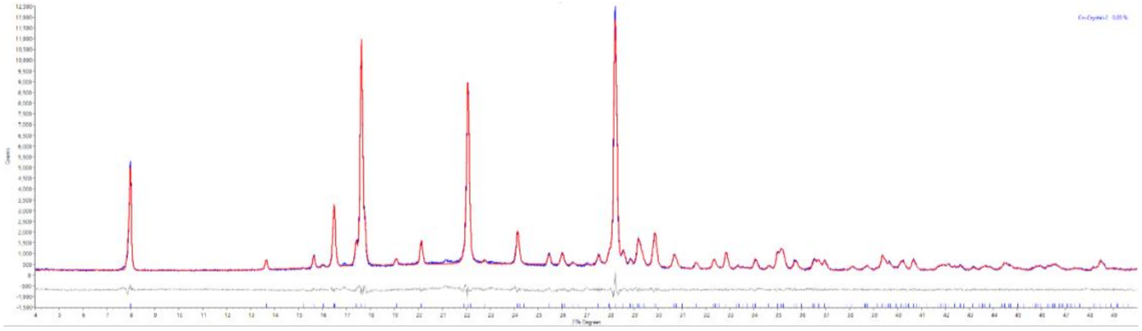

Figure S34. Pawley fit of PXRD pattern of **2** (sample soaked in water for for 6 h and heated at 80 ˚C for 5 mins), observed (blue), calculated (red) and difference plot [I_{obs}-I_{calc}] (grey) (2θ range 4-50 ˚). Refinement details **2**: Space group: *P-1*, Volume = 439.38(3) Å^3, a = 6.1112(2) Å, b = 7.0207(2) Å, c = 11.1217(4) Å, α = 67.912(2) ˚, β = 86.313(2) ˚, λ = 83.741(2) ˚, R_{wp} = 0.0661, $R_{wp'}$ = 0.1166, 173 parameters (9 background, 1 zero error, 5 peak profile, 6 lattice parameters and 152 reflections).

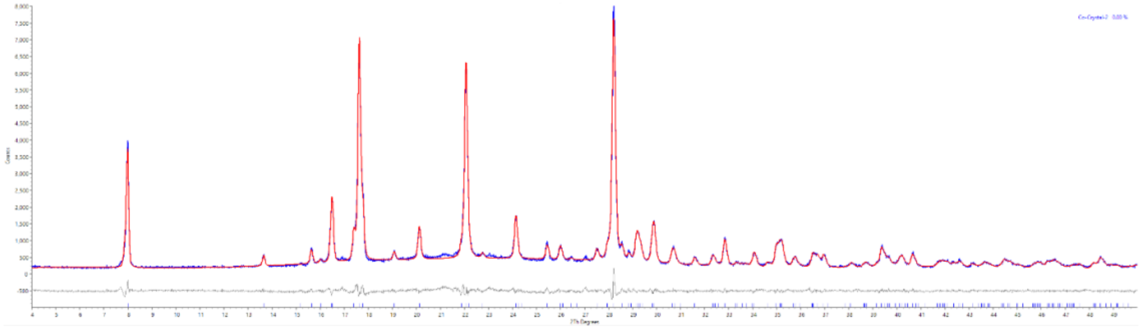

Figure S35. Pawley fit of PXRD pattern of **2** (sample sonicated in water for for 6 h and heated at 80 ˚C for 5 mins), observed (blue), calculated (red) and difference plot [I_{obs}-I_{calc}] (grey) (2θ range 4-50 ˚). Refinement details **2**: Space group: *P-1*, Volume = 439.63(3) Å^3, a = 6.1124(3) Å, b = 7.0227(3) Å, c = 11.1224(5) Å, α = 67.917(2) ˚, β = 86.311(3) ˚, λ = 83.733(2) ˚, R_{wp} = 0.0684, $R_{wp'}$ = 0.1266, 173 parameters (9 background, 1 zero error, 5 peak profile, 6 lattice parameters and 152 reflections).

Finally, the heating time was increased for both samples of **2**: (1) *soaked* and (2) *sonicated in water for 6 h before being heated at 80 ˚C for a total of 30 minutes*. In both instances, co-crystal **2** was retained, but there was also evidence of the 1,2-bis(4-pyridyl)ethylene (**bpe**) phase.

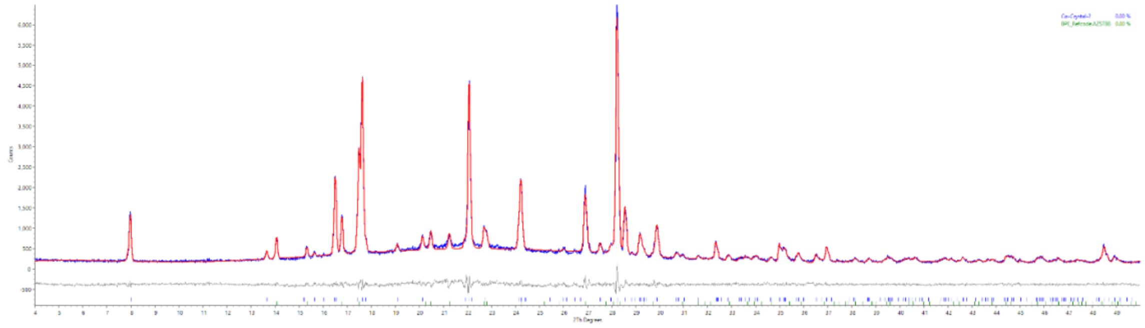

Figure S36. Two-phase Pawley fit of PXRD pattern of **2** and **bpe** (from sample of **2** soaked in water for 6 h and heated at 80 ˚C for 30 mins), observed (blue), calculated (red) and difference plot [I_{obs}-I_{calc}] (grey) (2θ range 4-50 ˚). Refinement details: **2** Space group: *P-1*, Volume = 438.83(4) Å^3, a = 6.1097(3) Å, b = 7.0156(3) Å, c = 11.1175(6) Å, α = 67.930(4) ˚,β = 86.318(4) ˚,λ = 83.725(3) ˚, **bpe** Space group: *P2₁/a*, Volume = 477.46(5) Å^3, a = 7.8373(5) Å, b = 10.5540(7) Å, c = 5.7784(4) Å, α = 90 ˚,β = 92.602(6)˚,λ = 90 ˚, R_{wp} = 0.0722, $R_{wp'}$ =0.1563, 264 parameters (9 background, 1 zero error, 9 peak profile, 10 lattice parameters and 235 reflections).

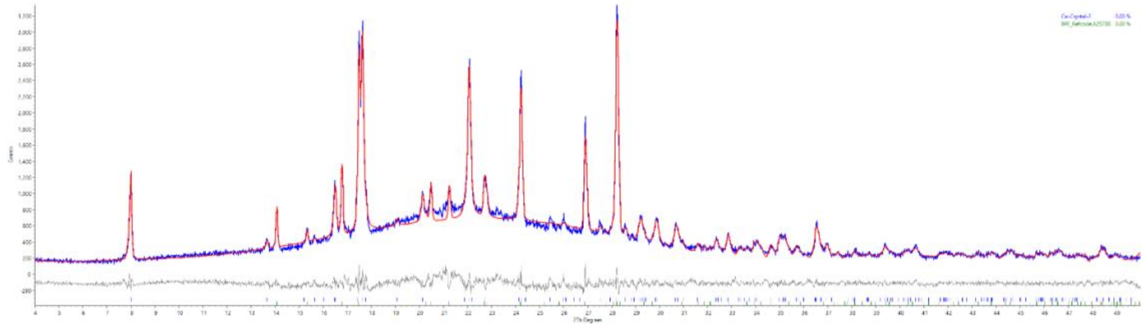

Figure S37. Two-phase Pawley fit of PXRD pattern of **2** and **bpe** (from sample of **2** sonicated in water for 6 h and heated at 80 ˚C for 30 mins), observed (blue), calculated (red) and difference plot [I_{obs}-I_{calc}] (grey) (2θ range 4-50 ˚). Refinement details of **2**: Space group: P-1, Volume = 439.50(8) Å^3, a = 6.1124(5) Å, b = 7.0220(7) Å, c = 11.123(1) Å, α = 67.876(7) ˚,β = 86.319(6) ˚,λ = 83.739(5) ˚, **bpe** Space group: *P2₁/a*, Volume = 478.59(7) Å^3, a = 7.8403(5) Å, b = 10.563(1) Å, c = 5.7849(5) Å, α = 90 ˚,β = 92.604(5) ˚,λ = 90 ˚, R_{wp} = 0.0738, $R_{wp'}$ = 0.1680, 264 parameters (9 background, 1 zero error, 9 peak profile, 10 lattice parameters and 235 reflections).

The thermal instability of **2** was further tested using a Linkam TC92 hot-stage microscope. Single crystals of **2** were soaked in water before being placed on a glass slide and placed on the heating stage of the microscope. Single crystals were heated to 80 ˚C, which resulted in the water evaporating and the surface of the crystals turning opaque. The crystals were, in turn, removed from the hot stage and covered in oil for further inspection. Despite the opaque powdery surface, colourless crystals were still present when the surface material was removed. A colourless crystal was then analysed by single-crystal diffraction. The data quality was poor in comparison to a pristine single crystal, but confirmed the crystal to be 1,2-Bis(4-pyridyl)ethylene (**bpe**). Summary details of the structure solution of **bpe** are outlined in **Table S2**.

Table S2. Summary details of structure solution of **bpe**, crystal isolated from thermal analysis of **2**.

	**bpe**<br>$C_{12}H_{10}N_2$<br>182.22
Crystal habit	block
Crystal colour	Colourless
Crystal size (mm)	0.07×0.04×0.04
Crystal system	monoclinic
Space group, Z	P21/c, 2
a (Å)	5.7283(7)
b (Å)	10.5510(14)
c (Å)	7.5830(12)
α (°)	90
β (°)	91.693(14)
γ (°)	90
V ($Å^3$)	458.11(11)
Density (g cm^{-3})	1.321
Temperature (K)	100.00(10)
μ (mm^{-1})	0.625
Radiation (λ)	Cu Kα (λ = 1.54184)
2θ range (°)	14.388 to 131.248
Reflns. collected	3148
Independent reflns. (R_{int})	755 (R_{int} =0.0663)
Reflns. used in refinement, n	755
LS parameters, p	64
Restraints, r	0
R1 (F)[a] I>2σ(I)	0.0765
wR2 (F^2)[a], all data	0.2095
S(F^2)[a], all data	1.017

[a]$R1(F) = \Sigma(|F_o| - |F_c|)/\Sigma|F_o|$; $wR2(F^2) = [\Sigma w(F_o^2 - F_c^2)^2/\Sigma wF_o^4]^{1/2}$; $S(F^2) = [\Sigma w(F_o^2 - F_c^2)^2/(n + r - p)]^{1/2}$

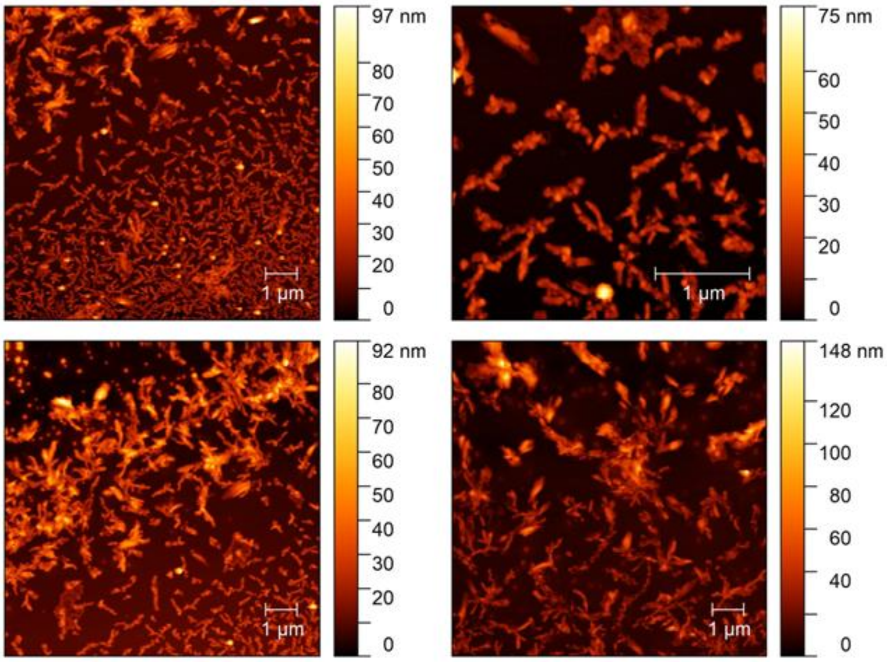

Figure S38. AFM images of the **F_4DBrB** suspension in water.

Unlike **XON1**, when bromo-nanosheet suspension was deposited on a zero background flat plate, a large number of crystals of **bpe-H_2O** was observed. When images were taken under

the microscope, these crystals appeared similar to the crystals obtained for deposition of only **bpe** suspension (Figure S22). This indicates **bpe** dissolution upon sonication of co-crystal **2**.

For suspension of **2**, we tried to quantify the material left in solution in a similar manner to **XON1**. Figure S39 shows the normalised absorption of exfoliated **2** before and after centrifugation with spectra for **bpe** and **F4DBrB**. In contrast to **XON1**, the spectra for exfoliated **2** showed a close match for **bpe**. Weak Tyndal scattering is still observed, but minimal absorbance at 800 nm indicates a lack of nanoparticles in suspension. **F_4DBrB** is not evident in the UV-vis spectra indicating it precipitates from solution so likely explains the weak Tyndal scattering observed for exfoliated **2** suspensions. We have therefore concluded that bromo-nanosheets fully dissolve.

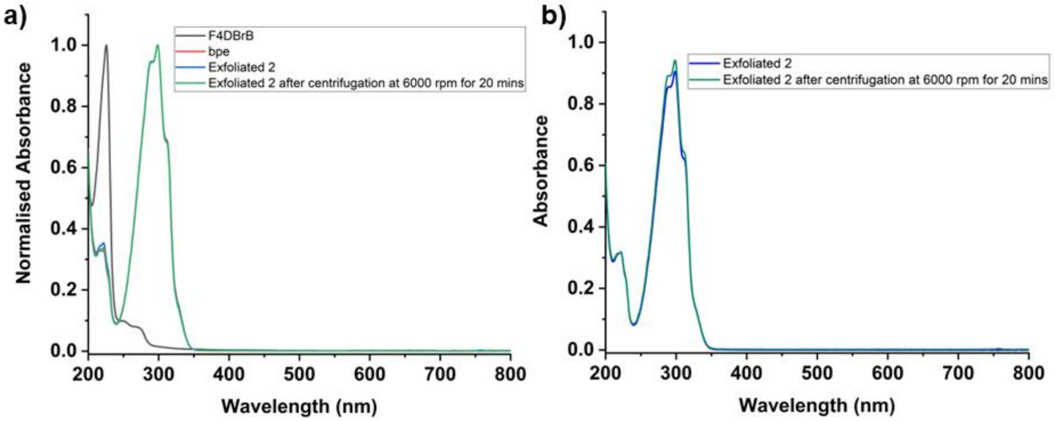

Figure S39. a) Normalised UV spectra of **bpe**, **F_4DBrB**, exfoliated **2** before and after centrifugation at 6000 rpm for 20 mins, b) UV-vis stack plot of exfoliated **2** suspension before and after centrifugation at 6000 rpm for 20 mins.

4. Stability test for Nanosheets

Thermal Stability

Nanosheet suspension of **XON1** was heated at 80 °C in sealed reaction vials. **XON1** showed strong Tyndall scattering after 2 days of heating. The **XON1** nanosheet was then re-isolated by centrifugation at 4500 rpm for 1 hour, drop-casted on a flat plate, then subjected to PXRD analysis (Figures 3a and S40). Powder diffraction analysis showed evidence of **XON1**.

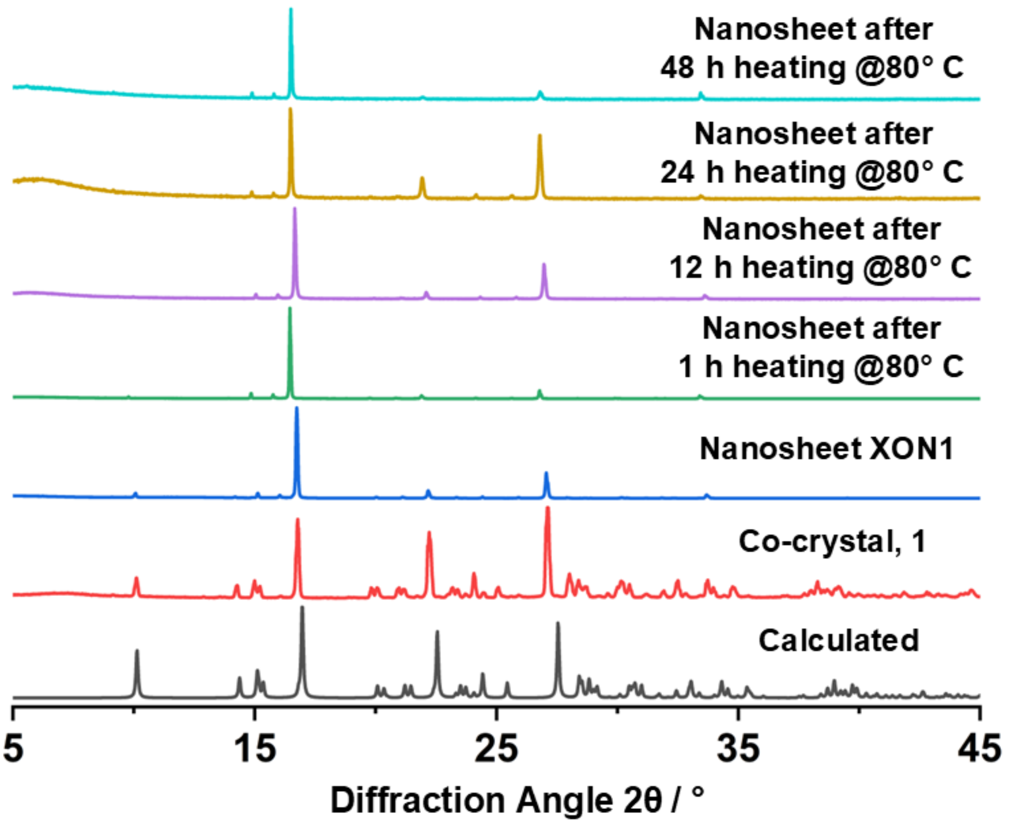

Figure S40. Powder X-ray diffraction patterns of **XON1** after heating at 80° C at different intervals. From bottom to top: the calculated pattern from room temperature single crystal data (ref code: QIHCAL03), as synthesised co-crystal, nanosheet **XON1**, nanosheet **XON1** after heating at 80° C for 1 h, 12 h, 24 h and 48 h.

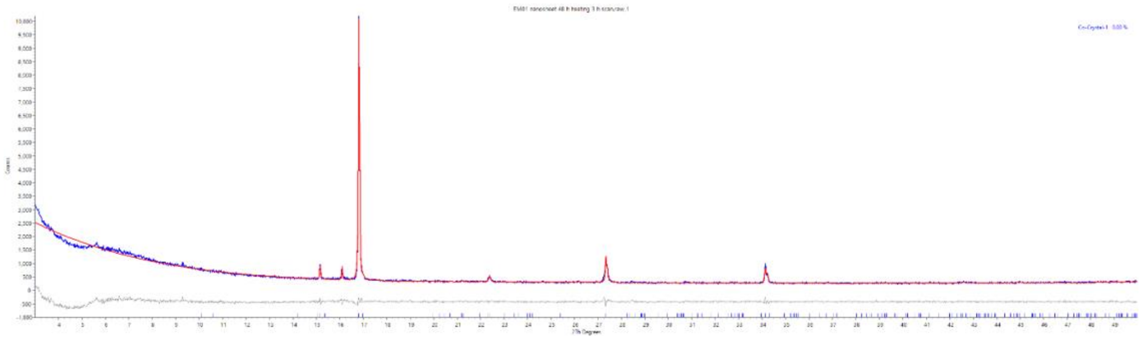

Figure S41. Pawley fit of PXRD pattern of Nanosheet **XON1** heated at 80° C for 48 h, observed (blue), calculated (red) and difference plot [I_{obs}-I_{calc}] (grey) (2θ range 4-50 ˚). Refinement details **1** Space group: *P-1*, Volume = 460.6(5) Å^3, a = 6.275(4) Å, b = 8.536(5) Å, c = 9.295(6) Å, α = 83.17(3) ˚, β = 70.96(2) ˚, λ = 78.63(3) ˚, R_{wp} = 0.0707, $R_{wp'}$ = 0.3631, 187 parameters (9 background, 2 radiation contamination, 1 zero error, 5 peak profile, 6 lattice parameters and 166 reflections).

pH Stability

The **XON1** nanosheet suspension was treated separately with aqueous solutions of $HCl_{(aq)}$ and $NaOH_{(aq)}$ of different pH values (pH 1, 4, 11 and 14). For each treatment, 2 mL of nanosheet suspension was placed in a glass vial followed by gradual addition up to 2 mL of a solution of a particular pH. The mixtures were shaken vigorously, and the Tyndall scattering was monitored (Figure S42). The same method was followed for all pH ranges.

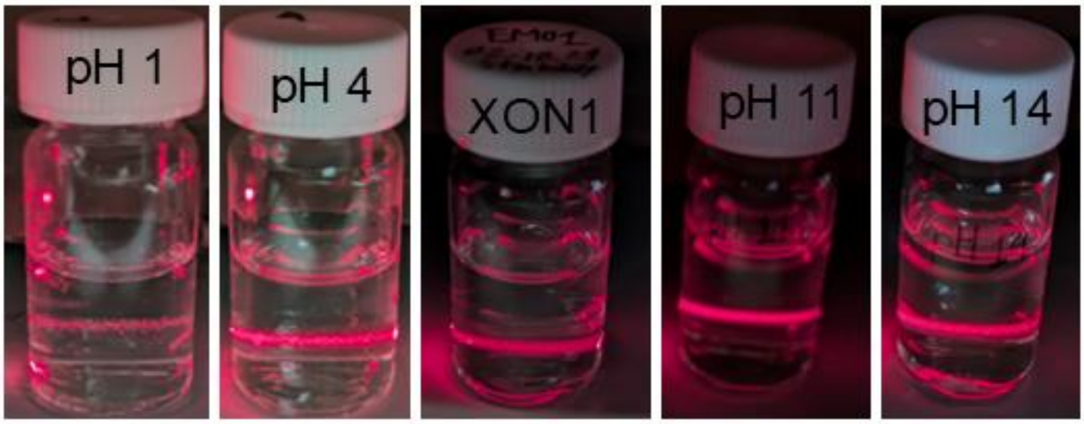

Figure S42. Tyndall scattering effects exhibited by suspensions of **1** after acid and base treatment.

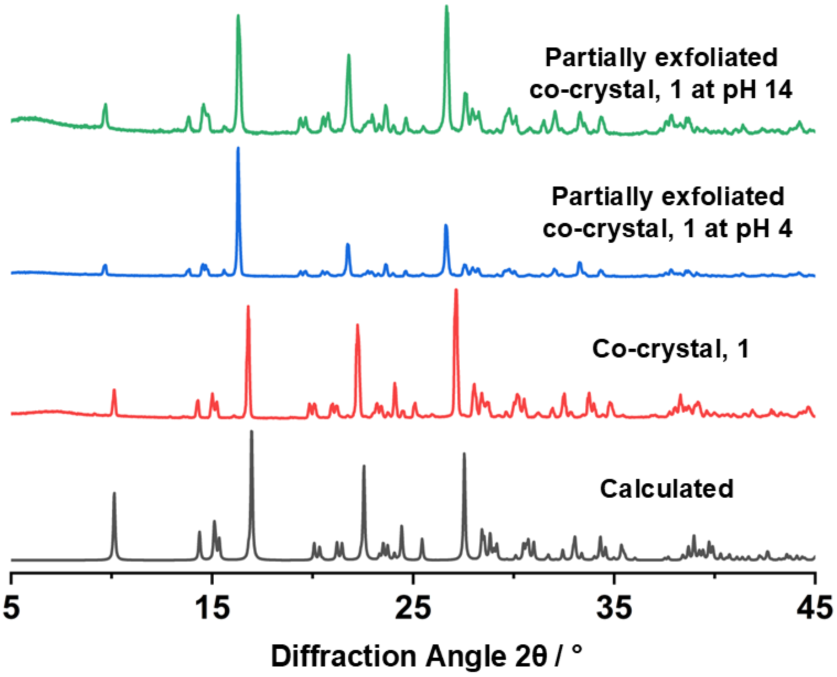

Figure S43. Powder X-ray diffraction patterns of **1** after acid treatment. From bottom to top: the calculated pattern from room temperature single crystal data (ref code: QIHCAL03), as-synthesised co-crystal, partially exfoliated co-crystal treated with $HCl_{(aq)}$ (pH4).

5. Binding energy calculation for Halogen-bonded Organic Nanosheets (XONs) and Hydrogen-bonded Organic Nanosheets (HONs)

Density functional calculations were carried out with CP2K software[11] using the PBE functional[12] with D3 dispersion correction,[13] with Goedecker–Teter–Hutter (GTH)

pseudopotentials[14] and localised double-ζ basis sets with diffuse and polarization functions (DZVP) optimised for use in CP2K[15] in combination with plane waves with a cutoff of 400 Ry.

All XONs and HONs systems were calculated as three-dimensional (3D) periodic systems. To describe one-dimensional (1D) and two-dimensional (2D) systems of XONs, the lattice vector in the non-periodic dimension was set to 30 Å, to ensure that there was at least 10 Å of free space between replicas in the non-periodic dimension. For HON two-dimensional (2D) systems, the lattice vector in the non-periodic dimension was set to 20 Å. Experimental X-ray crystal structures were used as the starting structures for both XONs and HONs. For XONs, all atom coordinates and unit cell parameters for 1D and 2D systems were fully optimised (apart from those in non-periodic directions), while cell parameters of the 3D bulk systems were kept at their experimental values (Figures S44-S47). For HONs, all atom coordinates were fully optimised, while cell parameters were kept at their experimental values (apart from those in non-periodic directions which were set to 20 Å).

Binding energies of 1D, 2D and 3D halogen-bonded systems were calculated relative to isolated molecules of 1,2-bis(4-pyridyl)ethylene (**bpe**) and 1,4-diiodotetrafluorobenzene (**F_4DIB**) for **1** or 1,2-bis(4-pyridyl)ethylene (**bpe**) and 1,4-dibromotetrafluorobenzene (**F_4DBrB**) for **2**:

$E_{binding} = E(1D/2D/3D) - E(bpe) - E(F_4DXB)$

Where *E(1D/2D/3D)* is the energy of the 1D, 2D or 3D iodine- or bromine-based system, *E(bpe)* is the energy of a single **bpe** molecule and *$E(F_4DXB)$* is the energy of a single **F_4DIB** or **F_4DBrB** molecule.

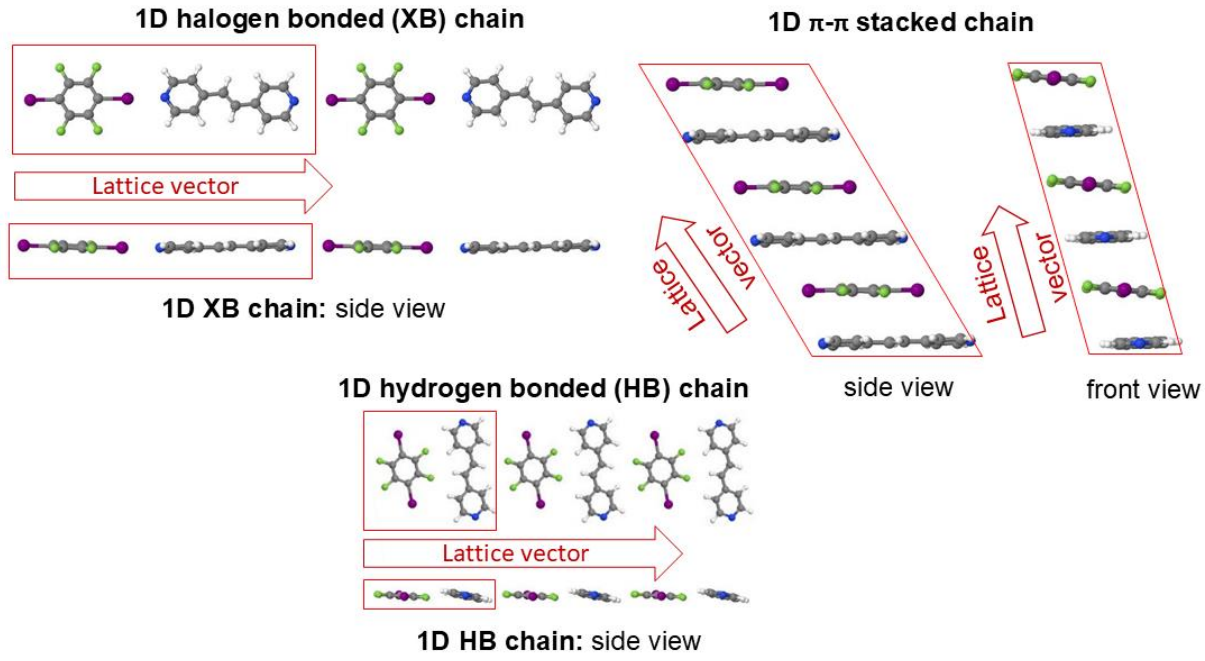

Figure S44. Calculated structures of 1D building blocks of **1**, showing the 1D halogen-bonded (XB), 1D π–π stacked and 1D hydrogen-bonded (HB) chains. Colour codes: C (grey), H (white), N (blue), F (green) and I (violet).

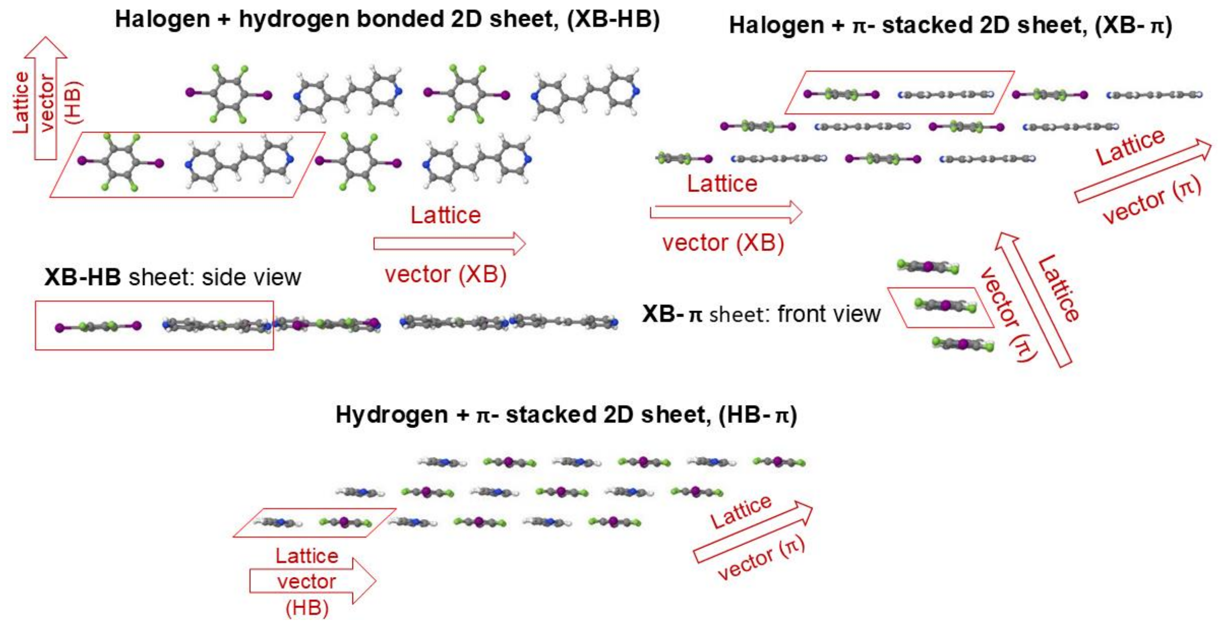

Figure S45. Calculated structures of 2D sheets of **1**, showing the 2D XB-HB (halogen-bonded and hydrogen-bonded) sheet, 2D XB-π (halogen-bonded and π–π) sheet and 2D HB-π (hydrogen-bonded and π–π) sheet. Colour codes: C (grey), H (white), N (blue), F (green) and I (purple).

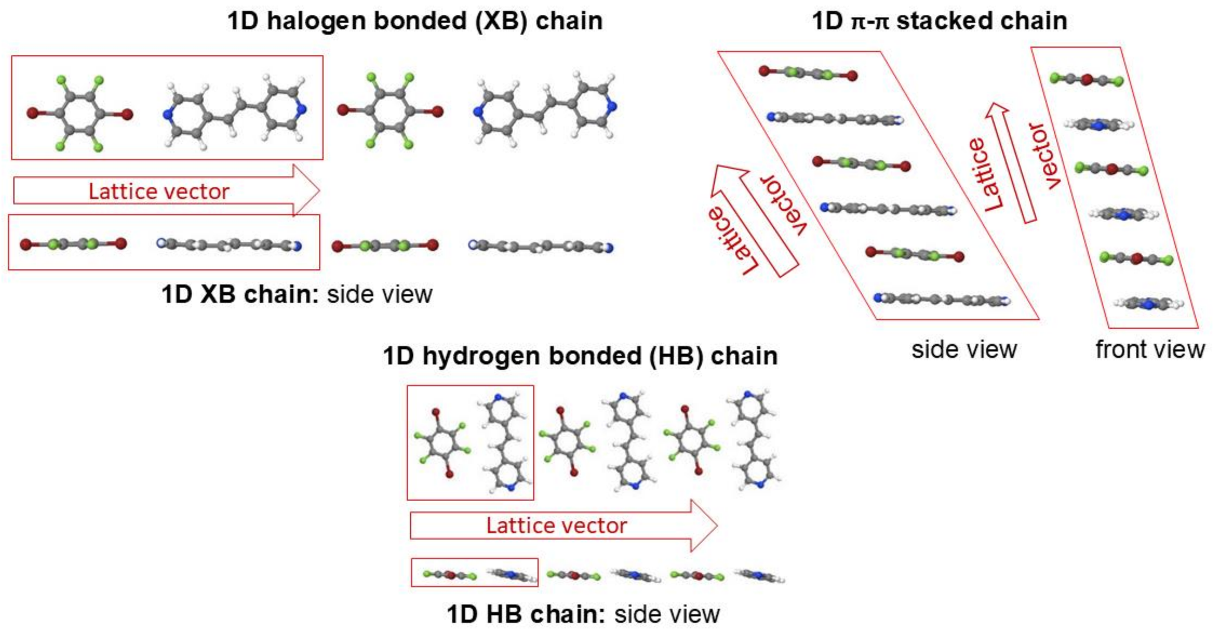

Figure S46. Calculated structures of 1D building blocks of **2**, showing the 1D halogen-bonded (XB), 1D π–π stacked and 1D hydrogen-bonded (HB) chains. Colour codes: C (grey), H (white), N (blue), F (green) and Br (brown).

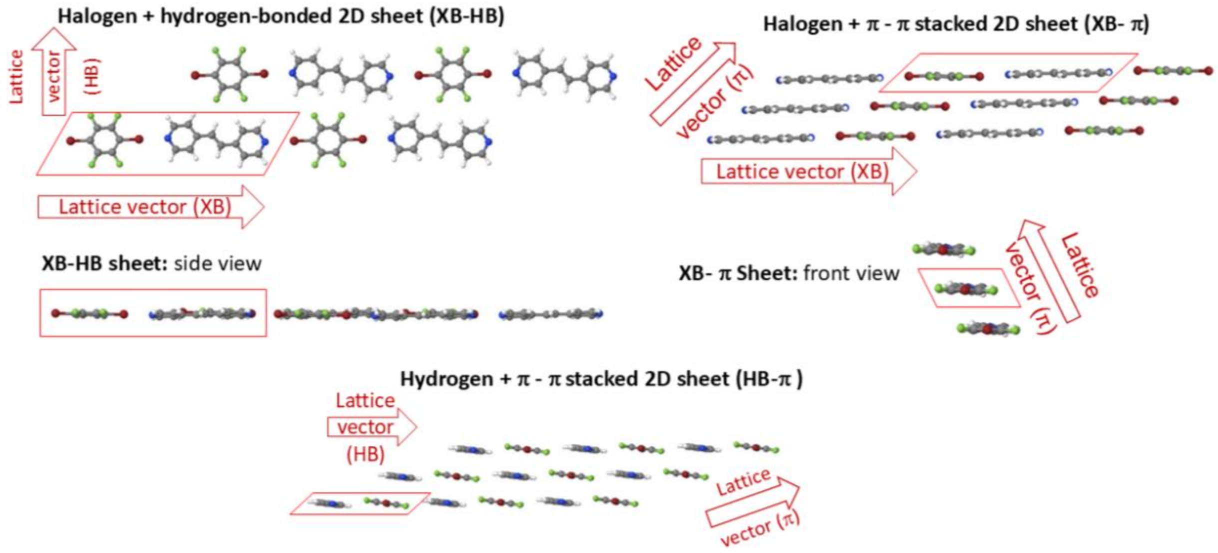

Figure S47. Calculated structures of 2D sheets of **2**, showing the 2D XB-HB (halogen-bonded and hydrogen-bonded) sheet, 2D XB-π (halogen-bonded and π–π) sheet and 2D HB-π (hydrogen-bonded and π–π) sheet. Colour codes: C (grey), H (white), N (blue), F (green) and Br (brown).

To compare binding energies of XONs with existing supramolecular nanosheets, we have calculated binding energies for our previously reported HONs: **HON1** and **HON2**. These HONs were prepared by ultrasonic liquid exfoliation of their layered counterpart HOF, **1** and HOF, **2.TP** respectively. The HOFs have strong charge assisted hydrogen bonds within the layers and π-π interactions in between the layers.

For HOF, **1** the binding energies are relative to a single molecule of **1**. For **2.TP**, the energies are relative to the pair of molecules (unit cell). Three lattice vectors A, B and C were considered. Vectors A, B and C were chosen to go from the 3D to the 2D structure.

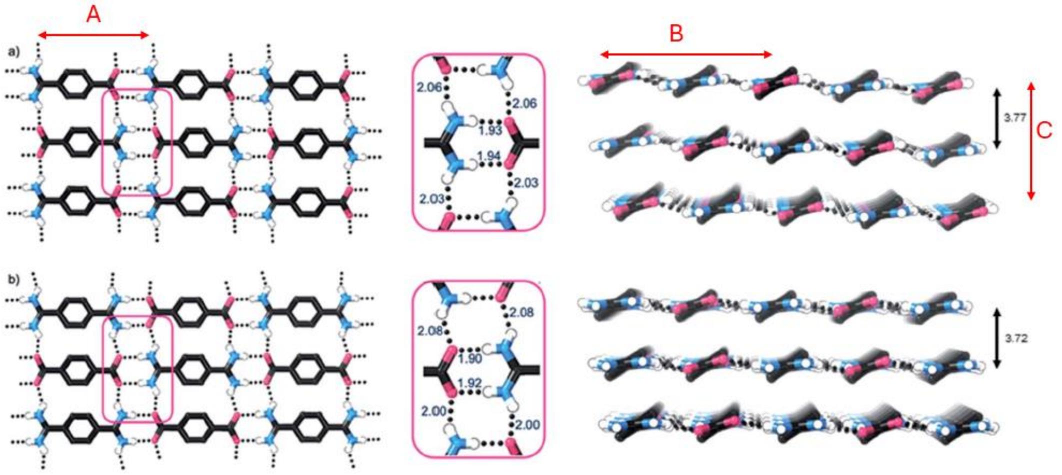

Figure S48. Structures[16] of 2D sheets of a) **HON1** and b) **HON2**, showing charge assisted hydrogen bonds in A and B directions and π-π stacking of the 2D sheets in C direction. Colour codes: C (black), H (white), N (blue) and O (pink).

Table S3. Calculated binding energies for 2D HONs based on experimental crystal structure.

Systems	Binding energies, kcal/mol		
	Lattice Vectors	1	2.TP
Two-dimensional hydrogen bonding	A, B	-72.11	-56.64

6. References

[1] G. S. Pawley, *J. Appl. Crystallogr.* **1981,** *14*, 357–361.
[2] A. A. Coelho, *Topas Acad. Version 7, see http//www.topas-academic.net*.
[3] A. A. Coelho, *J. Appl. Crystallogr.* **2018**, *51*, 210–218.
[4] CrysAlisPro 1.171.43.122a *(Rigaku Oxford Diffraction, 2024)*
[5] O. V. Dolomanov, L. J. Bourhis, R. J. Gildea, J. A. K. Howard and H. Puschmann, *J. Appl. Cryst.* **2009**, *42*, 339-341.
[6] G. M. Sheldrick, *Acta Crystallogr. Sect. C Struct. Chem.* **2015**, *71*, 3–8.
[7] D. Nečas, P. Klapetek, *Open Physics* **2012**, *10*, 181-188.
[8] (a) A. De Santis, A. Forni, R. Liantonio, P. Metrangolo, T. Pilati, G. Resnati, *Chem. Eur. J.* **2003**, *9*, 3974-3983; (b) A. Forni, P. Metrangolo, T. Pilati, G. Resnati, *Cryst. Growth Des.* **2004**, *4*, 291-295.
[9] D. J. Ashworth, A. Cooper, M. Trueman, R. W. M. Al-Saedi, L. D. Smith, A. J. H. M. Meijer, J. A. Foster, *Chem. Eur. J.* **2018**, *24*, 17986-17996.
[10] E. Zolotoyabko, *J. Appl. Crystallogr.* **2009**, *42*, 513-518.
[11] T. D. Kühne, M. Iannuzzi, M. Del Ben, V. V. Rybkin, P. Seewald, F. Stein, T. Laino, R. Z. Khaliullin, O. Schütt, F. Schiffmann, D. Golze, J. Wilhelm, S. Chulkov, M. H. Bani-Hashemian, V. Weber, U. Borštnik, M. Taillefumier, A. S. Jakobovits, A. Lazzaro, H. Pabst, T. Müller, R. Schade, M. Guidon, S. Andermatt, N. Holmberg, G. K. Schenter, A. Hehn, A. Bussy, F. Belleflamme, G. Tabacchi, A. Glöß, M. Lass, I. Bethune, C. J. Mundy, C. Plessl, M. Watkins, J. VandeVondele, M. Krack, J. Hutter, *J. Chem. Phys.* **2020**, *152*, 194103.
[12] J. P. Perdew, K. Burke, M. Ernzerhof, *Phys. Rev. Lett.* **1996**, *77*, 3865-3868.
[13] S. Grimme, J. Antony, S. Ehrlich, H. Krieg, *J. Chem. Phys.* **2010**, *132*, 154104.
[14] S. Goedecker, M. Teter, J. Hutter, *Phys. Rev. B* **1996**, *54*, 1703-1710.
[15] J. VandeVondele, J. Hutter, *J. Chem. Phys.* **2007**, *127*, 114105.
[16] J. Nicks, S. A. Boer, N. G. White, J. A. Foster, *Chem. Sci.* **2021**, *12*, 3322-3327.
